# Response of a Diverse European Soybean Collection to “Short Duration” and “Long Duration” Drought Stress

**DOI:** 10.3389/fpls.2022.818766

**Published:** 2022-02-17

**Authors:** Aamir Saleem, Jonas Aper, Hilde Muylle, Irene Borra-Serrano, Paul Quataert, Peter Lootens, Tom De Swaef, Isabel Roldán-Ruiz

**Affiliations:** ^1^Plant Sciences Unit, Flanders Research Institute for Agriculture, Fisheries and Food (ILVO), Merelbeke, Belgium; ^2^Department of Plant Biotechnology and Bioinformatics, Ghent University, Ghent, Belgium

**Keywords:** phenotypic diversity, breeding, *Glycine max*, growth rate, phenology, yield, UAV, growth curve

## Abstract

Drought causes significant damage to a high value crop of soybean. Europe has an increasing demand for soybean and its own production is insufficient. Selection and breeding of cultivars adapted to European growth conditions is therefore urgently needed. These new cultivars must have a shorter growing cycle (specifically for adaptation to North-West Europe), high yield potential under European growing conditions, and sufficient drought resistance. We have evaluated the performance of a diverse collection of 359 soybean accessions under drought stress using rain-out shelters for 2 years. The contrasting weather conditions between years and correspondingly the varying plant responses demonstrated that the consequences of drought for an individual accession can vary strongly depending on the characteristics (e.g., duration and intensity) of the drought period. Short duration drought stress, for a period of four to 7 weeks, caused an average reduction of 11% in maximum canopy height (CH), a reduction of 17% in seed number per plant (SN) and a reduction of 16% in seed weight per plant (SW). Long duration drought stress caused an average reduction of 29% in CH, a reduction of 38% in SN and a reduction of 43% in SW. Drought accelerated plant development and caused an earlier cessation of flowering and pod formation. This seemed to help some accessions to better protect the seed yield, under short duration drought stress. Drought resistance for yield-related traits was associated with the maintenance of growth under long duration drought stress. The collection displayed a broad range of variation for canopy wilting and leaf senescence but a very narrow range of variation for crop water stress index (CWSI; derived from canopy temperature data). To the best of our knowledge this is the first study reporting a detailed investigation of the response to drought within a diverse soybean collection relevant for breeding in Europe.

## Introduction

Drought can cause significant damage to crops and may compromise global food security (FAO, 2017). Important yield losses related to drought have been reported for different crops including wheat (27.5%), rice (25.4%), maize (14.0%), and soybean (21.8%; Zhang et al., 2018; Wang et al., 2020a). Main consequences of drought stress in crops are the reduction in leaf area and in stem elongation, which negatively affect the productivity (Mangena, 2018). To withstand drought conditions, plants can adopt a variety of strategies that involve morphological, physiological and biochemical responses, with considerable diversity observed among or even within crop species (Farooq et al., 2012). Important plant responses to drought are changes in stomatal regulation (Pirasteh-Anosheh et al., 2016), changes in the root system (Ye et al., 2018), hormonal changes (Kaur et al., 2016), activation of antioxidant defense systems (Sun et al., 2020) or osmotic adjustments (Turner, 2018). The nature and the magnitude of the crop responses to drought also depends on the duration (and the intensity) of the stress. For example, it has been shown that mild to moderate stress affects mainly stomatal functioning, while severe stress limits the photosynthesis mainly because of damage of the photosynthetic apparatus (Pirasteh-Anosheh et al., 2016; Wang et al., 2018). Timing of the stress situation is also important to consider. For example, if a period of drought occurs early in the season, the plants can compensate for negative effects when more favorable conditions return (Dong et al., 2019; Cui et al., 2020). In contrast, a period of stress at critical developmental phases can cause irreversible damage, leading to high crop productivity losses (Wei et al., 2018; Dong et al., 2019). Given the complexity and diversity of the adaptive mechanisms to drought, the development of drought resistant crops requires the consideration of multiple traits and responses to drought stress as well as their interactions.

Soybean [*Glycine max* (L.) Merr.] is the fourth leading crop worldwide, cultivated on over 120.5 million hectares (FAOSTAT, 2021). Europe is the second largest consumer of soybean after China. With continuously increasing demand, the area of soybean cultivation in Europe almost doubled from 2011 to 2018, up to 5.5 million hectares and 11.6 million tons produced in 2019 (FAOSTAT, 2021). Despite this fast increase in acreage and production volume, local soybean cultivation accounts for only 34% of the total 34.4 million tons consumed in Europe (IDH and IUCN NL, 2019; FAOSTAT, 2021). The selection of adapted varieties through breeding, is an essential step to end European dependence on imported soybean (Van Schoote, 2012; IDH and IUCN NL, 2019; Würschum et al., 2019; Boulch et al., 2021). The industry recognizes this need, with several European breeding programs already underway including those in Austria (Saatbau Linz), Belgium (Storm Seeds and Protealis), France (RAGT), Germany (IG Pflanzenzucht and University of Hohenheim), and Serbia (IFVCNS).

European agriculture is mainly rain-fed, with a share of irrigated area of only 6% (Rossi, 2019). Under the current scenario of changing climatic conditions, the frequency of dry spells associated with low rainfall and high temperature is expected to increase (UNDRR, 2021). Moreover, the period between seedling emergence and reproductive development in soybean can coincide with the occurrence of dry spells in many European regions, as observed in recent years (Bastos et al., 2020; Fu et al., 2020; Peters et al., 2020). This poses serious concerns for European soybean production. Considering the urgent need to expand the soybean cultivation area in Europe under changing climatic conditions, soybean varieties must be developed that are adapted to cultivation in Europe as well as robust to drought stress.

The first and most important step to improve drought resistance of soybean bred for European cultivation is to define effective selection criteria. It has been shown that soybean is only moderately sensitive to drought stress at the seedling stage because water demand is low at this phase in development (Wei et al., 2018; Dong et al., 2019). The developmental stages following flowering are more critical, where soybean plants require sufficient water to achieve maximum yield potential (Kranz and Specht, 2012). Investigating the effect of drought at reproductive stages is therefore considered to be more relevant in this crop (Do Rosário Rosa et al., 2020; Du et al., 2020; Yan et al., 2020). Drought stress at flowering and pod formation reduces the growth rate, leading to shorter plants (Wei et al., 2018); this reduction in plant height has been associated with a decrease in photosynthetic performance (Mak et al., 2014; Zhao et al., 2020). Therefore, the ratio of plant height under drought compared to well-watered conditions is frequently used to estimate the effect of drought (Wang et al., 2020b; Dhungana et al., 2021). Drought at reproductive stages can also cause earlier senescence and a reduction of the leaf area (Wei et al., 2018), which can have a negative impact on yield. Plants that display low leaf senescence (LSEN) under drought maintain a relatively high leaf water content and retain their photosynthetic activity (Rivero et al., 2007), which can be a yield-protecting mechanism. Another frequently investigated trait in soybean is canopy wilting, with slow wilting genotypes tending to be more resistant to drought because of a higher water use efficiency (Ries et al., 2012). Canopy wilting has been also related to canopy temperature, which provides an indirect measure of transpiration rate and stomatal conductance (Bai and Purcell, 2018). Soybean genotypes that utilize water more efficiently under drought conditions maintain a low canopy temperature and are considered more resistant than those generating higher canopy temperatures (Kaler, 2017; Kumar et al., 2017). Drought can also affect the phenology. For example, stress at the reproductive stage can shorten flowering and pod filling duration and increase the rate of senescence (Desclaux and Roumet, 1996). Evaluation of these varied and complex phenological responses is therefore important to understand the impact of drought stress on yield (Jumrani and Bhatia, 2018).

Manual measurements and visual observations are still broadly applied, although interest in the use of unmanned aerial vehicles (UAV) equipped with different imaging sensors for soybean phenotyping is on the rise. UAVs have been employed for quantification of wilting (Zhou et al., 2020), estimation of maturity stage (Yu et al., 2016; Zhou et al., 2019; Trevisan et al., 2020), quantification of plant density (Ranđelović et al., 2020) or leaf area index (Yuan et al., 2017), and prediction of yield (Yu et al., 2016; Zhang et al., 2019b; Herrero-Huerta et al., 2020; Maimaitijiang et al., 2020; Zhou et al., 2021). Similarly, in previous work (Borra-Serrano et al., 2020) we developed a UAV-based approach to estimate canopy cover and canopy height and to derive parameters related to growth and development in soybean. UAVs that capture images in a short time lapse allow for screening of a large number of plots under the same environmental conditions (e.g., temperature, wind, and light), which is an important advantage when estimating the plant responses to stress situations.

In light of the need to develop soybean cultivars adapted to cultivation in Europe and the anticipated increased risk of drought, the general aim of this study was to investigate how a broad set of soybean accessions relevant for breeding in Europe respond to drought conditions. This can inform soybean breeders about the most relevant characteristics to use in future breeding efforts. Specific objectives of this study were: (1) to describe the phenotypic diversity present in a diverse soybean collection and its potential for breeding efforts in Europe; (2) to evaluate the performance of this collection for traits related to growth and phenology under drought stress conditions, in relation to their performance under well-watered conditions; (3) to assess the “broad-sense” heritability of traits related to drought resistance in this diverse collection; and (4) to identify plant traits that can be considered by breeders to select for drought resistance in germplasm of relevance for Europe.

## Materials and Methods

### Plant Material

A set of 359 soybean accessions originating from 25 countries in Europe, China and the United States were used in this study. This subset of the “EUCLEG collection” described in Saleem et al. (2021) includes accessions of relevance for soybean breeding in Europe (Supplementary Table S1). The accessions were divided into four “growth groups” (GP), based on maturity information that was either publicly available at the start of this study, delivered by the provider of the seeds or generated in previous experiments. GP1 (*n* = 90), GP2 (*n* = 91), GP3 (*n* = 88), and GP4 (*n* = 90) comprised accessions expected to belong to maturity group MGI/II, MG0, MG00, and MG000, respectively. However, as the information available was rather limited and was not completely reliable in some cases, this division into groups does not correspond perfectly with the classification in maturity groups.

### Field Trials

**Figure 1:**
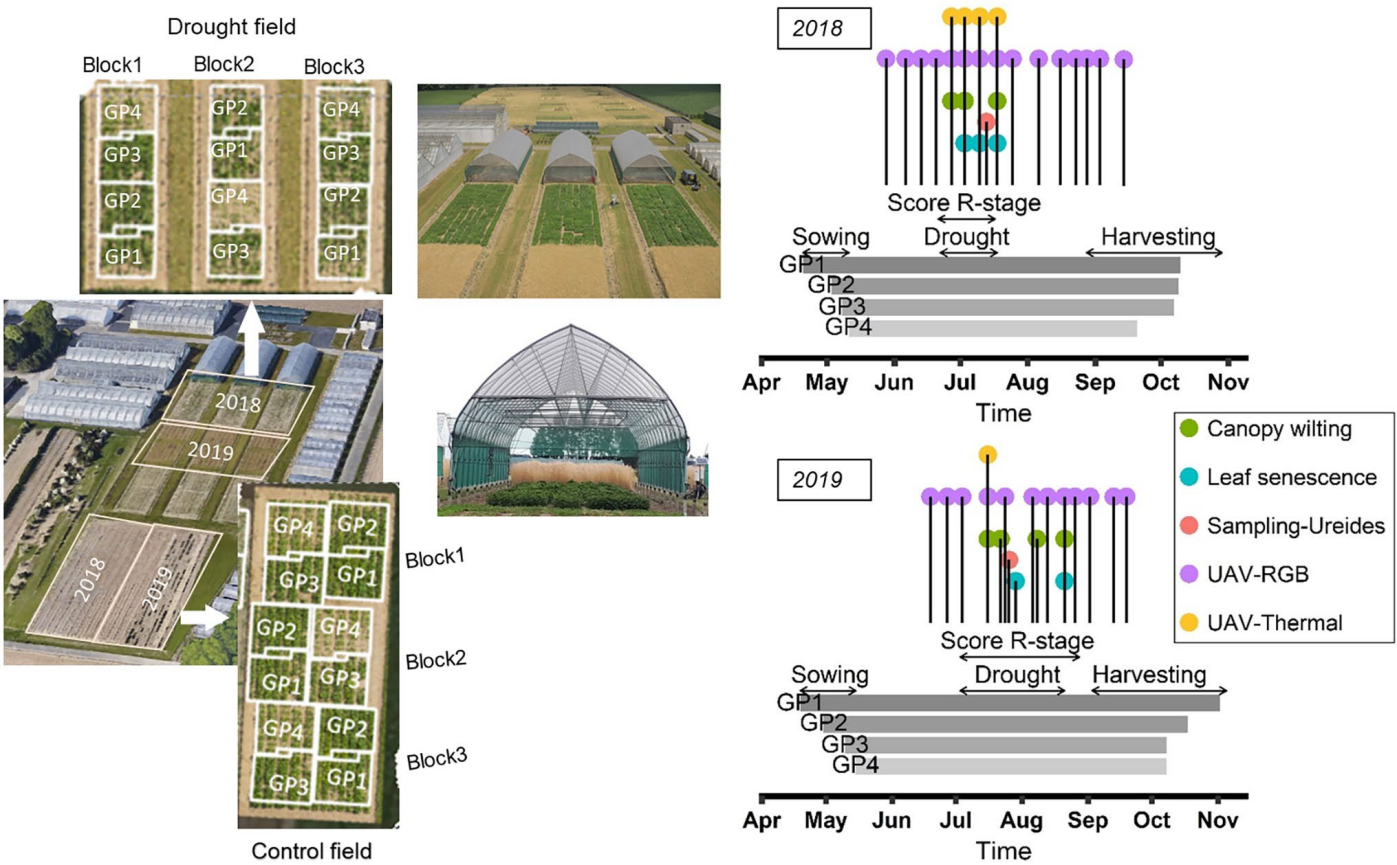
Field plans and timing of the observations. Left: layout of control and drought fields in 2018 and 2019, and general view of the rain-out shelters. Right: schematic representation of the timing of the observations performed in 2018 and 2019. Horizontal arrows delineate the following periods: sowing, determination of R-stage, drought treatment and harvesting. Vertical lines indicate the moments when canopy wilting and leaf senescence were scored, when samples were taken for the determination of ureides concentration, and the timing of the RGB and thermal UAV flights.

In 2018, the accessions were sown in two adjacent fields at the same location in Melle, Belgium (51.00° N, 3.80° E) on a sandy loam soil (Figure 1). One of these fields was used as “control” treatment under well-watered conditions, while the other was subjected to a drought treatment using rain-out shelters as described below. The design of each field was an augmented row-column design in which three check genotypes (one from each GP1, GP2, and GP4) were replicated nine times, nine check genotypes (two from each GP1, GP2, GP4, and three from GP3) were replicated six times, and 13 check genotypes (three from each GP1, GP3, GP4, and four from GP2) were replicated three times. The remaining 334 genotypes were not replicated. This resulted a total of 454 plots in the control treatment and 454 plots in the drought treatment. The check genotypes were well-characterized varieties (Supplementary Table S1). Two similar setups were established on adjacent fields at the same location in 2019 with the same set of soybean accessions (Figure 1). Adjacent fields were used in different years to avoid “legacy effects” of the previous soybean trial. The randomization scheme was different for each year.

To apply the recommended row-to-row distance and sowing density for genotypes of different maturity groups (45, 55, 65, and 75 seeds m^−2^ for MGI/II, MG0, MG00, and MG000, respectively), the plot dimensions were slightly adapted for the different GPs. Each plot comprised three rows. For GP1 and GP2, with row-to-row distance of 0.4 m, plot dimensions were 1.20 × 0.75 m (area 0.90 m^2^). For GP3 and GP4, with row-to-row distance of 0.25 m, plot dimensions were 0.75 × 1.20 m. The four GPs were sown on four different dates in an attempt to synchronize the developmental stage at which drought was imposed (i.e., when 50% of the plots had initiated flowering). Sowing dates in 2018 were 20 April, 2 May, 8 May, and 11 May for GP1, GP2, GP3, and GP4, respectively. In 2019 the sowing dates were 19 April, 30 April, 10 May, and 15 May for GP1, GP2, GP3, and GP4, respectively. These sowing dates were chosen using available data from previous soybean trials in which the optimum sowing time for accessions from different maturity groups had been determined for the study location (data not shown).

#### Trial Management and Drought Treatment

An overview of the different field activities is provided in Supplementary Table S2. In short, the seeds were inoculated with a commercial strain of *Bradyrhizobium japonicum* before sowing according to the manufacturer’s instructions. In 2018 the inoculant product was BIODOZ^®^ (De Sangosse, France) but as the same product was not available in 2019, seeds were inoculated with HiStick^®^ (BASF, United States). Both products have been shown to render sufficient nodulation in Belgium (Pannecoucque et al., 2018). Fertilizers were applied before sowing in both years and weeds were controlled by application of herbicides directly after sowing and after manual removal in June. After emergence, thinning was carried out to standardize the plant density to 60% of the total seeding density in GP1 (27 plants m^−2^) and GP2 (33 plants m^−2^), while no thinning was required on GP3 and GP4 plots. During the drought treatment, one insecticide application was necessary to control spider mites. Irrigation was applied as required, first manually with a hose at seedling stage and with sprinklers at later stages to maintain sufficient soil moisture in the drought and control fields until flowering had started on 50% of the plots. After that, a period of drought was imposed between 22 June and 18 July in 2018, and between 3 July and 21 August in 2019 to the plots of the drought field. This was achieved by placing mobile rain-out shelters over the drought field (Figure 1). Sprinkler irrigation was continued as required in the control field. In 2018 the soil moisture was monitored in the drought field before, during and after the drought treatment using 36 time domain reflectometer sensors (TDR; type CS616, Campbell Scientific, United Kingdom; 12 per rain-out shelter, Figure 1) distributed throughout the field at soil depth of 30 cm and connected with a data logger (CR1000, Campbell Scientific, United Kingdom). TDR measurements were performed only during and after the drought period in 2019. The drought treatment was maintained until the canopy wilting and LSEN symptoms as observed at noon became clearly visible in most of the plots. After the drought treatment, irrigation was resumed in the drought field and was continued until the late developmental stages in control and drought fields. Meteorological conditions were recorded using a temperature and relative humidity (RH) probe (CS215, Campbell Scientific, Inc., United Kingdom), a precipitation sensor (ARG100, Campbell Scientific, Inc., United Kingdom) and a pyranometer (LP02, Hukseflux, Netherlands), all connected to an automatic data logging system (CR1000, Campbell Scientific, Inc., United Kingdom) installed at the trial site. These data were used to calculate the cumulative water deficit (CWD) for the field trial as the accumulation of the difference between daily reference evapotranspiration (ET0 in mm) and precipitation (P in mm), starting at 1 April. ET0 was calculated using the *ET.PenmanMonteith* function of the R package *Evapotranspiration* (Guo et al., 2020). The long-term CWD statistics for the region were calculated using combined weather data sets (from 1979 to 2021) from the Joint Research Centre (JRC MARS Meteorological Database) and the Royal Meteorological Institute (KMI).

#### Measurements

Figure 1 summarizes the time schedule of the different measurements in 2018 and 2019; Table 1 describes the traits that were determined. Whenever possible, observations were made in the middle row of each plot.

**Table 1:** Description of the traits determined in 2018 and 2019.

Group	Trait^a^	Description	Method of determination
Derived from manual measurements/visual scores	E	Percentage of seedlings emerged	Expressed as a percentage of the number of seeds sown.
	PLV	Plant length up to the second node (cm)	Determined with a scale in three representative plants per plot. Average value considered. 2018: 28 May (GP1), 1 June (GP2), 5 June (GP3) and 8 June (GP4); 2019: 17 June (GP1), 20 June (GP2), 20 June (GP3) and 2 July (GP4).
	R2	Thermal time from sowing to full flowering (GDD)	From the growth curve fitted for R-stage as a function of thermal time using the sigmoid function from Yin et al. (2003).
	R5	Thermal time from sowing to beginning seed (GDD)	From the growth curve fitted for R-stage as a function of thermal time using a sigmoid function from Yin et al. (2003).
	R8	Thermal time from sowing to pod maturity (GDD)	Observed in the field when 95% of pods per plot reach mature pod color.
	R2R5	Duration of pod formation (GDD)	Determined as the difference between R2 and R5.
	R5R8	Duration of seed development (GDD)	Determined as the difference between R5 and R8.
	R2R8	Thermal time from full flowering to pod maturity (GDD)	Determined as the difference between R2 and R8.
	CW	Canopy wilting (score 1–9; low-high)	2018: 27 June, 3 July, 17 July; 2019: 17 July, 22 July, 8 August, 21 August.
	LSEN	Leaf senescence (score 1–9; low-high)	2018: 4 July, 10 July, 20 July; 2019: 29 July, 21 August.
	PPS	Number of pods on the main stem	Determined on five plants per plot. Average value considered.
	SN	Number of seeds per plant	Determined on five plants per plot. Average value considered.
	SW	Seed weight per plant (g)	Determined on five plants per plot. Average value considered.
UAV-RGB	CC75	Thermal time to canopy cover 75% (GDD)	Thermal time from sowing to canopy cover of 75%. Derived from the fitted growth curve of RGB canopy cover data.
	AGRmax	Maximum absolute growth rate (cm GDD^-100^)	Maximum rate of increase in canopy height. Derived from the fitted growth curve of RGB canopy height data.
	CH	Maximum canopy height (cm)	Maximum canopy height reached by the plot. Derived from the fitted growth curve of RGB canopy height data.
	DET	Degree of indeterminacy (GDD)	Duration of growth after initiation of flowering. Derived from the fitted growth curve of RGB canopy height data as the difference between thermal time to start of flowering and thermal to maximum canopy height.
	SNC	Rate of senescence (0–1)	Represents the rate of plant maturation at the end of cycle. Derived from the fitted growth curve of RGB canopy cover data as the difference between maximum canopy cover and the average lowest cover detected before the end of season.
UAV-Thermal	CWSI	Crop water stress index (0–1)	Index to quantify crop water stress, derived from thermal data according to De Swaef et al. (2021).

The traits have been grouped in three categories according to the mode of determination.

a For the determination of R2, R5, and R8 a score of developmental stage was assigned to each plot according to the scale of Fehr and Caviness (1977) during regular visits to the field (between 21 June and 17 July in 2018 and between 3 June and 27 June in 2019). R2R5, R5R8, and R2R8 were determined after correcting for spatial and residual variation in R2, R5, and R8. For the determination of PPS, SN, and SW five plants were collected on the middle row of each plot, bagged and transported to the laboratory.

##### Visual Measurements

Plant emergence was determined as the percentage of emerged plants in each plot. Plant length up to the second node (PLV) was measured at vegetative stage (between V2 and V5, where V2 and V5 are defined as the stages when a soybean plant displays two and five fully expanded trifoliate leaves, respectively; Fehr and Caviness, 1977). The reproductive stages (R-stages) from R1 to R8 were visually assessed regularly (one to two times per week), using the scale of Fehr and Caviness (1977). For example, R1 corresponds to the time when 50% of the plants in a plot have started flowering. R8 is the stage when 95% of the plants in a plot have reached mature pod color. Typically, the reproductive stage in function of time follows a double sigmoid pattern (R1 to R6 and R6 to R8; Setiyono et al., 2007), and is affected by temperature. Because it was not possible to visualize all R stages for all plots at the time of scoring, R stage data from R1 to R6 was used to fit a growth curve for the R stage as a function of thermal time (growing degree days: GDD) using the sigmoid function from Yin et al. (2003). Thermal time to full flowering (R2) and thermal time to the beginning of seed formation (R5) were then derived from the fitted curve. R2, R5, and R8 data were further used to calculate three other variables after correcting for spatial and residual differences: (1) duration of pod formation (R2R5) as the difference between R2 and R5, (2) duration of seed development (R5R8) as the difference between R5 and R8, and (3) total duration of reproductive development (R2R8) as the difference between R2 and R8. Canopy wilting (CW) was scored during the drought treatment three times in 2018 and four times in 2019. LSEN was scored during and at the end of the drought periods in 2018 and 2019. At the end of the growing period, five plants in the middle row of each plot were harvested and transported to the laboratory for the determination of the number of pods on the main stem (PPS), the number of seeds per plant (SN) and seed weight per plant (SW). The average values of five plants per plot were used for analysis.

##### Ureides Analysis

It has been demonstrated that drought stress causes an increase in the concentration of ureides in the stems of soybean plants (King and Purcell, 2005; Ray et al., 2015; Cerezini et al., 2017). We therefore checked whether the drought treatment had an effect on the physiology of the plants by quantifying the concentration of ureides in the stem under control and drought conditions. Four plants per plot were collected from the complete set of accessions from control and drought treatments on 13 July 2018 (3 weeks after initiation of the drought treatment) and transported to the laboratory for further processing. In 2019, plant samples were collected on 26 July (3 weeks after initiation of the drought treatment) from a subset of 40 plots representing 40 accessions (9 GP1, 7 GP2, 8 GP3, and 16 GP4) from both control and drought treatments. This subset was chosen as it represented the total genetic variation of the ureides concentration in the 2018 experiment. The samples were prepared according to the protocol of Unkovich et al. (2008). Leaves were removed and the stem fraction (including the petioles) was oven-dried for 2 days at 70°C. The dry material was ground first into coarse particles using the Peppink 200 AN Mill (Peppink, Netherlands) and then into fine particles using the Ball Mill MM 400 (Retsch, Germany). A homogeneous subsample of 100 mg was taken for the extraction and quantification of ureides according to Coleto et al. (2014).

##### UAV Measurements

The method developed by Borra-Serrano et al. (2020) was used to determine extra traits using UAV based approaches. RGB images (camera ɑ6000, Sony Corporation, Japan) were taken using a drone (model Onyxstar HYDRA-12, AltiGator, Belgium) during 15 flights between 28 May and 14 September 2018 and 12 flights between 19 June and 19 September 2019 (Figure 1). The thermal time needed for the canopy to cover 75% of the soil (CC75), the maximum absolute growth rate (AGRmax), the maximum canopy height (CH), the degree of indeterminacy (DET) and the rate of senescence (SNC) were determined for both years from these images as described in Borra-Serrano et al. (2020). In addition, plant canopy temperature was assessed four times in 2018 (between 26 June and 28 July) and once in 2019 (16 July) using a thermal camera (Wiris 2nd gen, Workswell, Czech Republic) mounted on a drone (model Onyxstar HYDRA-12, AltiGator, Belgium in 2018; model DJI Matrice 600 Pro, DJI, China in 2019). Thermal images were preprocessed in ThermoFormat (Workswell, Czech Republic) and then stitched in Agisoft Photoscan v.1.2.6 Professional Edition (Agisoft LLC, Russia). Canopy temperature data was extracted in QGIS v3.10 (QGIS Geographic Information System; Open Source Geospatial Foundation Project)^1^ with polygons defined over the middle row of each plot and only considering the pixels that correspond to vegetation as selected using a vegetation index. Air temperature, recorded at a 10 min interval by an automatic weather station (CR1000, Campbell Scientific, United Kingdom) installed at the experimental site, was used for computing mean ambient temperature for the period of 10:00 to 14:00 h. The crop water stress index (CWSI) was derived from the canopy temperature data according to De Swaef et al. (2021).

### Data Analysis

The data was first filtered for plots with more than 30% emergence and then cleaned for outliers according to Tukey’s rule (Tukey, 1977). As the treatments in 2018 and 2019 were different (see Results below), a separate analysis per year was carried out. The distribution of the data was inspected using Q-Q plots built in R version 3.6.3 (R Core Team, 2019) and, where necessary, a transformation was applied (indicated in Supplementary Table S3). The filtered and cleaned data were then analyzed using mixed linear models with the *lme4* package in R (Bates et al., 2015). The following base model was considered:


(1) Y=Genotype+Block+Column+Row

Where, “*Y*” is response variable, “*Genotype*” is random effect representing the accession, and “*Block*”, “*Column*,” and “*Row*” are random effects representing spatial components in the experimental design.

The base model was not applied as such because it would be “overfitted” (it incorporates the “*Block*” and “*Column*” as unique components while in the trial design columns were actually nested in the blocks). For each response variable the six versions of the base model were tested and the output was evaluated using the Akaike Information Criterion (AIC; Akaike, 1974). The best model was then chosen based on the lowest AIC value (Supplementary Table S3). From the best model, the best linear unbiased predictor value (BLUP) was calculated for each accession as the sum of the “*Intercept*” value and the value of the random effect of “*Genotype*”. Broad sense heritability (H^2^) was calculated from the variance components of the best model as follows:


(2) $$H^{2}=\frac{Var\left(Genotype\right)}{Var\left(Genotype\right)+Var\left(Residuals\right)}$$

For the interpretation of the heritability values we followed criteria as in Khan et al. (2020): low <30%; medium >30 and < 60%; and high >60%.

Summary statistics of BLUP values including the minimum, maximum, mean and coefficient of variation (CV) for each variable were calculated for the full collection and for each GP separately. To study the stability of the performance of the accessions across 2 years under well-watered conditions, Pearson’s correlations between the data of 2018 and 2019 from the control fields were calculated. Pearson’s correlations were also calculated between control and drought treatments for each year separately. For the traits that were determined several times each season (CWSI, CW, and LSEN), Pearson’s correlations between different time points were calculated.

To determine the response of the accessions to drought stress, a drought index (Yr) was calculated for the variables measured in both treatments (control and drought), according to Araghi and Assad (1998) as follows:


(3) $$Yr=\frac{Control-Drought}{Control}$$

Where,

“Yr” = 0: equal value in control and drought.

“Yr” > 0: lower value in drought than in control.

“Yr” < 0: higher value in drought than in control.

To determine the extent of variation in the response to drought for the measured traits, the coefficient of variation (CV) of the Yr values was calculated. Pearson’s correlations between Yr data and control treatment data were calculated in order to check for possible dependencies between the performance of the accessions under well-watered conditions and the strength of response to drought stress. A Principal Component Analysis (PCA) was performed to uncover patterns in this dataset. We used the R software package *factoextra 1.0.7.* (Kassambara and Mundt, 2020). The results were represented using a biplot that combines score and loading plots in a single graph highlighting the most prominent patterns of variation. To simplify the analysis and to get a more accurate representation of the general trends in the data sets, we first identified highly correlated variables that could be removed without loss of information. For this purpose, separate PCAs were carried out with subsets of variables [i.e., variables describing developmental responses (R2-Yr, R5-Yr, R8-Yr, R2R5-Yr, R5R8-Yr, and R2R8-Yr), growth related responses (AGRmax-Yr, CH-Yr, and SNC-Yr), traits of drought resistance (CW-Yr, LSEN-Yr, and CWSI-Yr), and yield responses (PPS-Yr, SN-Yr, and SW-Yr)].

Pearson’s correlations between Yr data of 2018 and 2019 were calculated to check the stability of how the accessions responded to drought in the 2 years investigated.

## Results

### Weather Conditions and Characteristics of the Drought Treatments in 2018 and 2019

The weather conditions in 2018 and 2019 are summarized in Supplementary Figure S1 and Supplementary Table S3. In 2018, the daily average temperature at the time of sowing was slightly higher than in 2019. During the vegetative and reproductive development of the crop, daily average temperature and daily solar radiation were also relatively higher in 2018 than in 2019 with the exception of a small period of 3 days at the end of July 2019 when the temperature rose above the critical threshold of 35°C. Also, at the time of harvesting, the average temperature was higher and the relative humidity was lower in 2018 than in 2019.

At the start of the drought treatment (when the rain-out shelters were placed over the plots) the average soil moisture content was 0.11 v/v in 2018 and 0.12 v/v in 2019 (Supplementary Figures S1C,D). The soil moisture content then dropped to an average of 0.06 v/v and 0.05 v/v in 2018 and 2019, respectively. Because of the fast response of the accessions to decreasing levels of soil moisture in 2018 shown as clearly visible symptoms of canopy wilting and LSEN, the rain-out shelters were removed after a period of drought treatment of 3–4 weeks and the soil moisture content was replenished to an average of 0.20 v/v.

Visual symptoms of drought stress developed more slowly in 2019. This was probably due to lower air temperature, lower solar radiation and higher air relative humidity (Supplementary Table S4), causing lower evapotranspiration as compared to 2018. These differences are also reflected in the cumulative water deficit index (CWD; Supplementary Figure S2). As the moment at which the drought treatment was applied was not exactly the same in the two seasons, it is not easy to compare the plots of 2018 and 2019 in Supplementary Figure S2. We see however that the CWD entered the “orange area” (representing the characteristics of “a one in 20 years” season) sooner after imposing the drought treatment in 2018 than in 2019, what might explain the quicker development of visual drought symptoms in 2018. Therefore, after a 6–7 week period of drought treatment in 2019, the rain-out shelters were removed and the soil moisture content was replenished to an average 0.14 v/v, a value which was considered sufficient at that time because most of the accessions had already progressed to advanced stages of reproductive development (many accessions were already at R6 stage) and thus required less water than the earlier reproductive stages as described by Tacker and Vories (2000).

The above results clearly show that the characteristics of the season and of the drought treatment were different in 2018 and 2019. The drought treatment was shorter in 2018, because the plants quickly developed visual symptoms of drought stress (canopy wilting or LSEN). In 2019 the drought treatment lasted longer and the plants only displayed visual symptoms of stress after several weeks of soil water depletion. In what follows, we use the terms “short duration drought stress” and “long duration drought stress” to refer to the drought treatments of 2018 and 2019, respectively.

### Overall Characteristics of the Data

**Figure 2:**
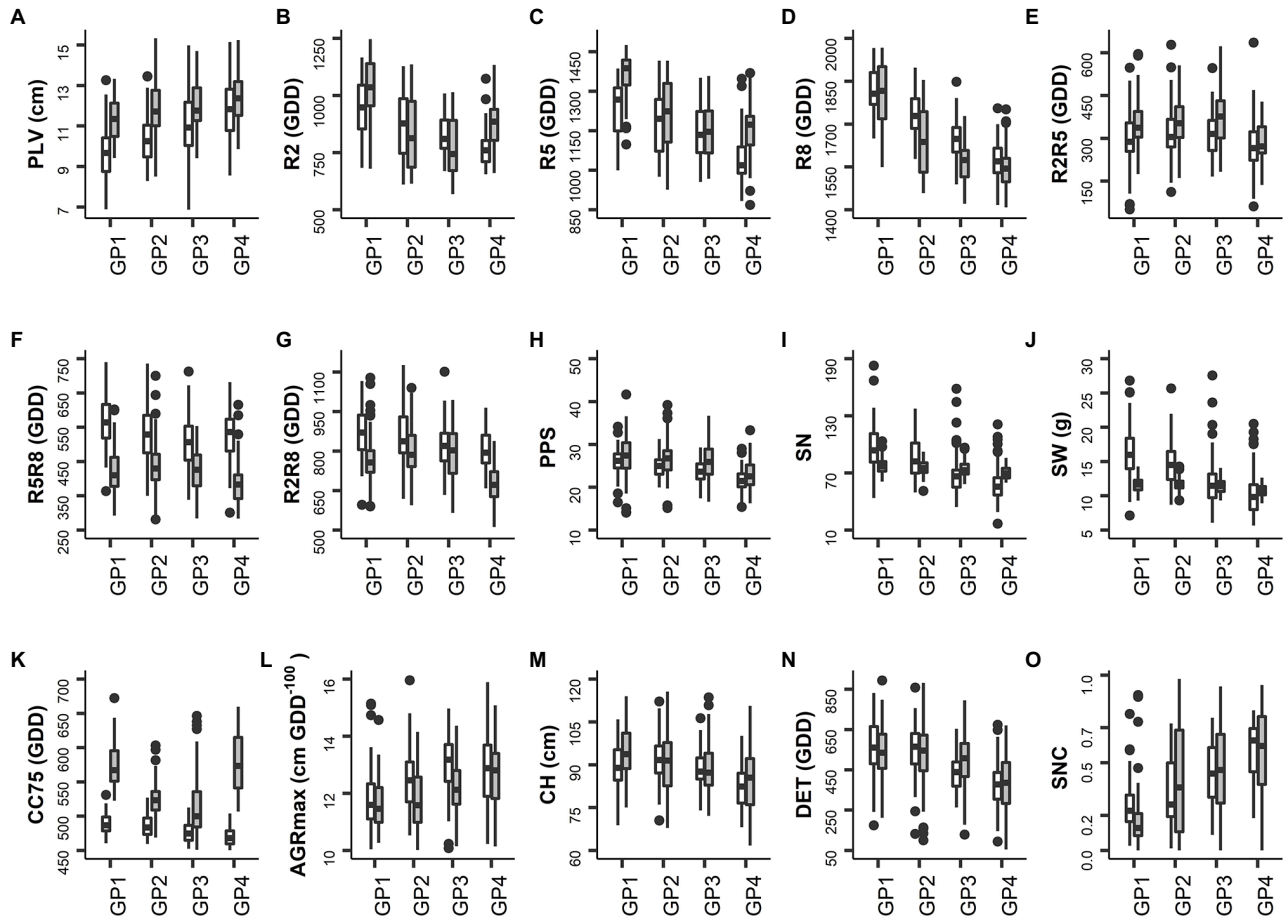
Distribution of the BLUP values per trait in the control field. White and gray shaded whiskers represent values for 2018 and 2019, respectively. Labels on the *X*-axis represent the four groups of accessions with different sowing moments (for details see Materials and Methods). **(A)** PLV: Plant length up to the second node. **(B)** R2: Thermal time from sowing to full flowering. **(C)** R5: Thermal time from sowing to beginning seed. **(D)** R8: Thermal time from sowing to pod maturity. **(E)** R2R5: Duration of pod formation. **(F)** R5R8: Duration of seed development. **(G)** R2R8: Thermal time from full flowering to pod maturity. **(H)** PPS: Number of pods on main stem. **(I)** SN: Number of seeds per plant. **(J)** SW: Seed weight per plant. **(K)** CC75: Thermal time to canopy cover 75%. **(L)** AGRmax: Maximum absolute growth rate. **(M)** CH: Maximum canopy height. **(N)** DET: Degree of indeterminacy. **(O)** SNC: Rate of senescence.

Seedling emergence (E) was variable in both years, with some plots displaying extremely low values. To avoid any bias in the results that might be caused by this, plots with *E* < 30% were not included in the analysis (for 2018, 29 and 32 plots were removed in control and drought fields, respectively, and for 2019, 30 and 68 plots were removed in control and drought fields, respectively). Considering the different characteristics of 2018 and 2019 (see above), we processed the data for each year separately. Linear mixed models were used to correct for spatial gradients (Supplementary Table S3) and BLUP values were calculated (Table 2; Figure 2).

**Table 2:** Summary statistics of BLUP values from control and drought fields in 2018 and 2019.

Trait^a^	Treat^b^	2018					2019				
		nObs	Mean ± SD	CV %	*H* ^2^ ^c^	Yr (Mean ± SD)^d^	nObs	Mean ± SD	CV %	*H* ^2^ ^c^	Yr (Mean ± SD)^d^
PLV (cm)	C	334	10.77 ± 1.60	14.86	0.76		331	11.90 ± 1.31	11.01	0.64	
	D	326	10.70 ± 1.90	17.80	0.81		303	11.68 ± 1.19	10.19	0.63	
R2 (GDD)	C	336	853.62 ± 123.96	14.52	0.82	0.00 ± 0.08 (317)	336	880.47 ± 158.30	17.98	0.97	0.09 ± 0.06 (300)
	D	330	852.54 ± 129.22	15.20	0.92		308	789.72 ± 138.10	17.49	0.95	
R5 (GDD)	C	321	1145.71 ± 122.89	10.73	0.74	0.03 ± 0.08 (298)	320	1208.68 ± 132.50	10.96	0.92	0.09 ± 0.06 (287)
	D	322	1101.46 ± 104	9.44	0.73		308	1098.99 ± 129.81	11.81	0.91	
R8 (GDD)	C	336	1690.18 ± 118.15	6.99	0.86	0.00 ± 0.04 (317)	336	1639.16 ± 134.07	8.18	0.94	0.04 ± 0.04 (297)
	D	330	1687.91 ± 110.49	6.55	0.86		305	1563.77 ± 90.38	5.78	0.75	
R2R5 (GDD)	C	311	300.09 ± 80.63	26.87		0.09 ± 0.31 (278)	315	341.18 ± 80.61	23.63		0.09 ± 0.19 (282)
	D	310	252.25 ± 62.52	24.80			306	309.31 ± 78.76	25.46		
R5R8 (GDD)	C	317	534.98 ± 77.88	14.56		−0.11 ± 0.17 (281)	315	413.17 ± 63.41	15.35		−0.16 ± 0.19 (275)
	D	311	583.90 ± 65.88	11.30			298	466.42 ± 64.99	13.93		
R2R8 (GDD)	C	331	834.20 ± 83.56	10.02		0.00 ± 0.08 (305)	331	754.30 ± 98.97	13.12		−0.03 ± 0.10 (291)
	D	324	831.40 ± 81.73	9.83			305	777.74 ± 104.02	13.37		
PPS (−)	C	333	24.01 ± 3.08	12.83	0.39	−0.02 ± 0.22 (311)	328	25.66 ± 4.32	16.84	0.56	0.26 ± 0.13 (283)
	D	327	24.62 ± 6.47	26.30	0.71		297	18.50 ± 2.14	11.57	0.38	
SN (−)	C	332	75.78 ± 23.49	31.00	0.67	0.17 ± 0.30 (307)	316	73.95 ± 7.59	10.26	0.32	0.38 ± 0.07 (277)
	D	323	64.76 ± 31.90	49.30	0.86		300	45.34 ± 3.93	8.67	0.22	
SW (g)	C	329	13.07 ± 3.83	29.30	0.66	0.16 ± 0.29 (305)	321	11.33 ± 1.02	9.00	0.27	0.43 ± 0.06 (273)
	D	321	11.05 ± 4.77	43.20	0.87		294	6.44 ± 0.42	6.52	0.16	
CC75 (GDD)	C	297	477.96 ± 17.32	3.62	0.32		305	545.70 ± 47.04	8.62	0.63	
	D	223	453.03 ± 63.49	14.00	0.75		274	600.89 ± 43.15	7.18	0.46	
AGRmax (cm GDD^−100^)	C	332	12.51 ± 1.24	9.91	0.51	0.22 ± 0.08 (292)	322	12.07 ± 1.09	9.03	0.47	0.22 ± 0.09 (274)
	D	307	9.70 ± 0.43	4.43	0.24		295	9.42 ± 1.07	11.11	0.38	
CH (cm)	C	315	87.57 ± 7.97	9.10	0.53	0.11 ± 0.11 (270)	315	89.14 ± 10.23	11.48	0.68	0.29 ± 0.08 (260)
	D	292	78.00 ± 11.39	14.10	0.83		280	62.75 ± 3.65	6.35	0.41	
DET (GDD)	C	321	473.8 ± 145.04	30.61	0.61		313	485.60 ± 157.42	32.42	0.73	
	D	293	530.65 ± 95.23	18.00	0.42		288	488.42 ± 48.19	9.87	0.28	
SNC (−)	C	309	0.40 ± 0.20	50.00	0.67	−0.26 ± 0.42 (218)	306	0.41 ± 0.29	70.73	0.9	−1.10 ± 1.33 (223)
	D	238	0.52 ± 0.20	38.50	0.67		282	0.69 ± 0.24	34.78	0.79	

Only traits that were determined during or after the drought treatment are considered. “nObs” is the number of observations after removal of plots with *E* < 30% and outliers, “Treat” is the treatment, “SD” is standard deviation, “CV %” is % genotypic coefficient of variation and “*H*^2^” is the broad sense heritability. PLV: Plant length up to the second node; R2: Thermal time from sowing to full flowering; R5: Thermal time from sowing to beginning seed; R8: Thermal time from sowing to pod maturity; R2R5: Duration of pod formation; R5R8: Duration of seed development; R2R8: Thermal time from full flowering to pod maturity; PPS: Number of pods on the main stem; SN: Number of seeds per plant; SW: Seed weight per plant; CC75: Thermal time to canopy cover 75%; AGRmax: Maximum absolute growth rate; CH: Maximum canopy height; DET: Degree of indeterminacy; SNC: Rate of senescence.

a R2R5, R5R8, R2R8 were determined from R2, R5 and R8 data after correcting for residual variation.

b ”C” represents the control treatment and “D” represents the drought treatment.

c *H*^2^ values were not calculated for R2R5, R5R8, and R2R8 as no variance components were estimated.

d Figure between brackets indicates the number of common genotypes observed in control and drought treatments. Yr values for PLV and CC75 were not considered as these traits correspond to moments before the initiation of drought period. Yr for DET was not calculated as it is more related to the growth habit of accessions and not directly linked to a response to drought.

For most traits, the level of variation observed in 2018 and 2019 in the control fields was similar. Exceptions were traits related to yield (SN and SW), which exhibited low variation in 2019 as compared to 2018. The levels of variation were also comparable between control and drought treatments for most of the traits with the exception of yield-related traits (PPS, SN, and SW) recorded in 2018 for which the variation was higher in the drought field than in the control field (Table 2), indicating a substantial response of the accessions to drought. In 2019 a strong overall reduction of the level of variation for yield-related traits (SN and SW) was observed, which was more pronounced in the drought field (Table 2).

The broad-sense heritability (*H*^2^) was high (0.73–0.94) in both years and in both treatments for phenological traits including R2, R5, R8, and SNC (Table 2). For yield-related traits *H*^2^ was medium to high (for PPS 0.38–0.71) or low to high (for SN and SW 0.16–0.87). Similarly, medium to high H^2^ values were obtained for CC75, CH, and DET (0.32–0.83), and low to high *H*^2^ for AGRmax (0.24–0.51). For LSEN, *H*^2^ was low at the start of the drought treatment, but it increased to values higher than 0.5 at later phases (Table 3). Similarly, *H*^2^ varied strongly between different dates of observation for CW (0.16–0.51). For CWSI, *H*^2^ was relatively high in the control (0.24–0.48) as compared to the drought field (0.01–0.13). These low *H*^2^ values in the drought fields can be explained by low variance observed in CWSI as discussed below.

**Table 3:** Summary statistics of BLUP values from control and drought fields in 2018 and 2019.

Trait	Year	Date^a^	Treatment	nObs	Mean ± SD	CV %	*H* ^2^	Yr (Mean ± SD)^b^
LSEN	2018	DAT-12	Drought	319	2.80 ± 0.20	7	0.28	
		DAT-17	Drought	321	3.32 ± 0.33	9.82	0.41	
		DAT-28	Drought	315	4.08 ± 0.52	12.87	0.56	
	2019	DAT-27	Drought	281	3.04 ± 0.08	2.62	0.13	
		DAT-50	Drought	283	4.20 ± 0.68	16.1	0.54	
CW	2018	DAT-5	Drought	329	4.18 ± 0.35	8.46	0.5	
		DAT-11	Drought	326	5.10 ± 0.43	8.37	0.36	
		DAT-25	Drought	306	3.04 ± 0.30	9.7	0.41	
	2019	DAT-15	Drought	308	2.32 ± 0.33	14.1	0.35	
		DAT-20	Drought	307	4.78 ± 0.42	8.8	0.28	
		DAT-37	Drought	302	3.56 ± 0.24	6.8	0.16	
		DAT-50	Drought	308	5.38 ± 0.79	14.7	0.51	
CWSI	2018	DAT-4	Control	319	0.17 ± 0.03	17	0.48	−2.0 ± 0.54 (280)
			Drought	304	0.50 ± 0.01	1	0.06	
		DAT-10	Control	331	0.21 ± 0.02	10.23	0.32	−2.54 ± 0.37 (307)
			Drought	323	0.73 ± 0.01	1.44	0.13	
		DAT-17	Control	327	0.36 ± 0.01	4.15	0.22	−0.43 ± 0.06 (300)
			Drought	323	0.51 ± 0.00	0	0.01	
		DAT-26	Control	330	0.13 ± 0.01	9	0.42	−3.89 ± 0.46 (295)
			Drought	310	0.64 ± 0.00	0	0.07	
	2019	DAT-13	Control	326	0.09 ± 0.01	8.69	0.24	−3.21 ± 0.36 (282)
			Drought	298	0.39 ± 0.00	1.5	0.08	

“nObs” is the number of observations after removal of plots with *E* < 30% and outliers, “SD” is standard deviation, “CV %” is % genotypic coefficient of variation and “*H*^2^” is the broad sense heritability. LSEN: Leaf senescence; CW: Canopy wilting; CWSI: Crop water stress index.

a “DAT” is days after treatment initiation.

b Figure between brackets indicates the number of common genotypes observed in control and drought treatments. Yr was not calculated for LSEN and CW as they were determined only in the drought field.

### Performance Under Well-Watered Conditions

Before analyzing the response of this soybean collection to drought, we investigated the overall performance of the accessions in the control treatment during the two seasons investigated.

#### General Trends

On average, the plants were slightly taller in 2019 than in 2018, as reflected in the values of PLV and CH (Table 2), but for CH this was particularly the case for GP1 and GP4 (Figure 2M). The thermal time required by the different GPs to achieve the development to full flowering (R2), seed formation (R5) and pod maturity (R8) in 2018 was in accordance with expectations, with GP1 requiring the highest number of GDD and GP4 the lowest. Correspondingly, the AGRmax increased from GP1 to GP4 (Figure 2L). In 2019 accessions of GP4 displayed some delay in development, reaching the R2 and R5 stages later than those of GP3 (Figures 2B,C). Delayed emergence of some accessions and leaf damage to several plots caused by rodents in 2019 (illustrated by the higher values of CC75 and lower values of AGRmax in 2019, Table 2) can explain this. The values of R2 and R5 were higher in 2019 than in 2018 (Table 2), indicating a slower path of vegetative development in 2019 and up to the initiation of seed formation. This trend was then reversed, with faster progress to pod maturity (lower value of R5R8 in 2019 than in 2018, Table 2). This can be explained by a short spell of high temperature that occurred between R5 and R8 as explained above. With some exceptions, these trends were also found when the different GPs were considered (Figures 2B–D).

Regarding traits directly linked to seed yield, there was a decreasing trend for values of PPS, SN, and SW from GP1 (late maturing accessions) to GP4 (early maturing accessions; Figures 2H–J). On average, the plants produced a similar number of pods on the main stem (PPS) in both years, but the seed number (SN) and the seed weight per plant (SW) were both lower in 2019 than in 2018 in the late maturing accessions (GP1 and GP2; Figures 2I,J). Also the range of variation for SN and SW was rather low in 2019 (Figures 2I,J). There seems to be a tendency for a higher level of indeterminacy (DET) in late maturing accessions (GP1) than in early maturing ones (GP4; Figure 2N). Differences between years for this trait were only marginal. As expected, the average rate of senescence (SNC) increased from GP1 to GP4 in both years (Figure 2O).

#### Correlation Between Traits and Between Years

Correlations between traits were generally low, except for traits that describe similar aspects, with the highest correlations among R2, R5, R8, and SNC and among PPS, SN, and SW (Supplementary Figure S3). Similar trends were found in both years under study, with only a few exceptions. Maximum canopy height (CH) was correlated positively with R2, R5, R8, PPS, SN, and SW in 2018 and 2019, confirming that late maturing accessions grew taller and produced more pods than early maturing accessions. Yield-related traits (PPS, SN, and SW), pod formation duration (R2R5) and seed development duration (R5R8) were not significantly correlated or the correlation coefficient was low. This indicates that in this set of accessions the length of these developmental phases (pod formation and seed development) does not linearly associate with seed yield.

In both years, the degree of indeterminacy (DET) correlated negatively with maximum absolute growth rate (AGRmax), showing that determinate accessions attain higher growth rates than indeterminate accessions. DET correlated positively with R2R5 and R2R8, confirming the expected relationship between degree of indeterminacy and duration of reproductive development.

The highest inter-year correlation values were obtained for R2, R5, and R8 (*R* = 0.62–0.8; Supplementary Figure S4). Correlation values for other traits including PLV, R2R8, PPS, CH, DET, and SNC were moderate (*R* = 0.5–0.6), while the inter-year correlation was low (*R* < 0.4) for R2R5, R5R8, SN, SW, CC75, and AGRmax.

### Drought Stress Responses

The drought treatment was imposed each year at the start of flowering in approximately 50% of the plots in the drought and control fields (Supplementary Figure S5). In 2018 the R stage for accessions of different GPs was similar when the drought treatment was initiated, but in 2019 accessions of GP2 and GP3 were more advanced, especially those in the drought field (Supplementary Figure S5). Sequential sowing of different GPs (see Materials and Methods section) was therefore quite successful for synchronizing the developmental stage of the different accessions up to initiation of the drought treatment, but some differences were still present. As anticipated, the concentration of ureides was higher in the drought field than in the control field in all the accessions in 2018, as well as in most of the accessions sampled in 2019 (Supplementary Figure S6). This revealed that the imposed treatment clearly caused a stress condition.

**Figure 3:**
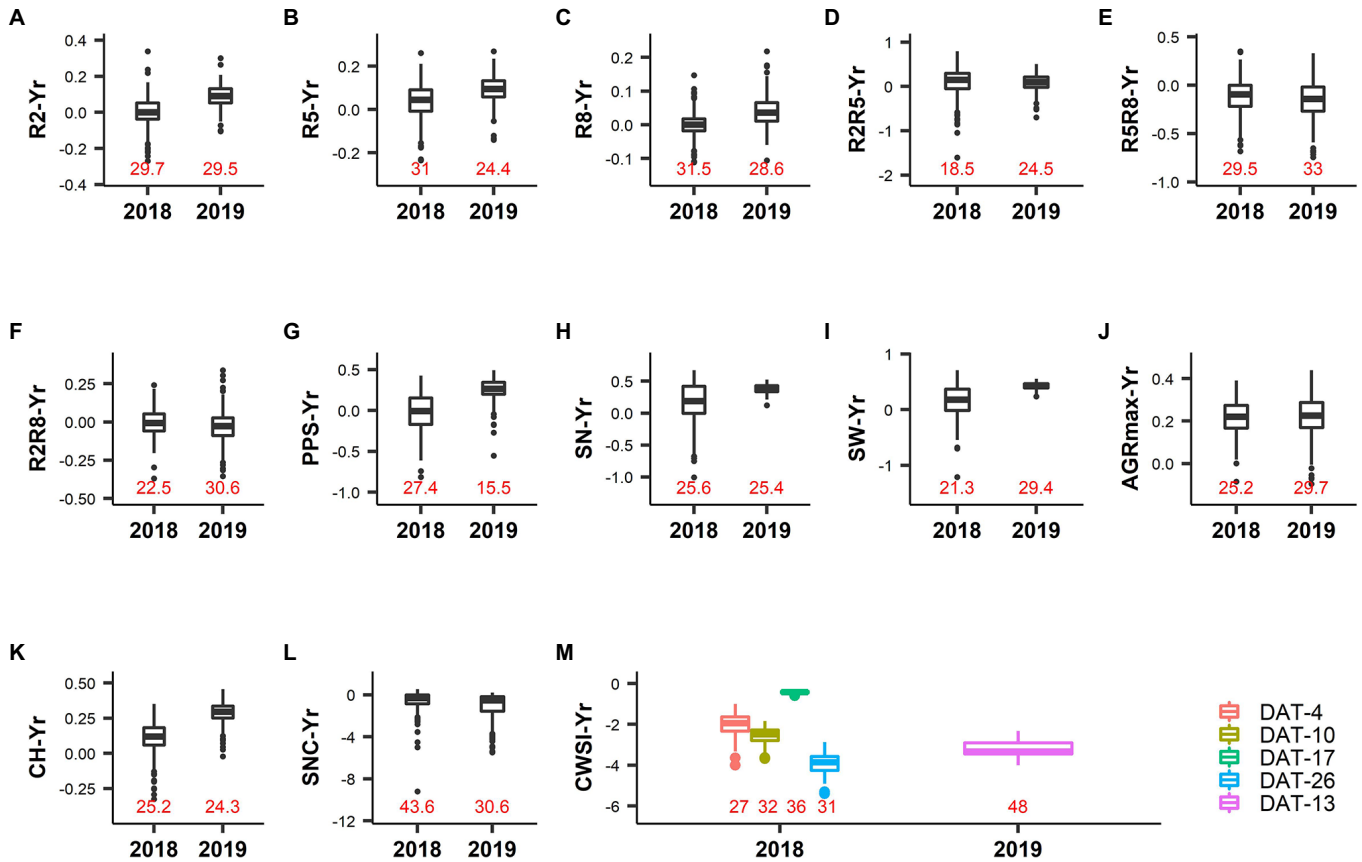
Phenotypic variation in drought index values (Yr) of different traits. Labels on the *X*-axis represent the year of the experiment, and correspond to a short drought treatment (2018) and a long drought treatment (2019). The *Y*-axis represents the drought index value of the respective trait. Data labels inside the plot area (colored in red) indicate the value of the coefficient of variation determined from the normalized Yr data. **(A)** R2: Thermal time from sowing to full flowering. **(B)** R5: Thermal time from sowing to beginning seed. **(C)** R8: Thermal time from sowing to pod maturity. **(D)** R2R5: Duration of pod formation. **(E)** R5R8: Duration of seed development. **(F)** R2R8: Thermal time from full flowering to pod maturity. **(G)** PPS: Number of pods on the main stem. **(H)** SN: Number of seeds per plant. **(I)** SW: Seed weight per plant. **(J)** AGRmax: Maximum absolute growth rate. **(K)** CH: Maximum canopy height. **(L)** SNC: Rate of senescence. **(M)** CWSI: Crop water stress index. Legends represent the measurement day after drought treatment initiation (DAT).

**Figure 4:**
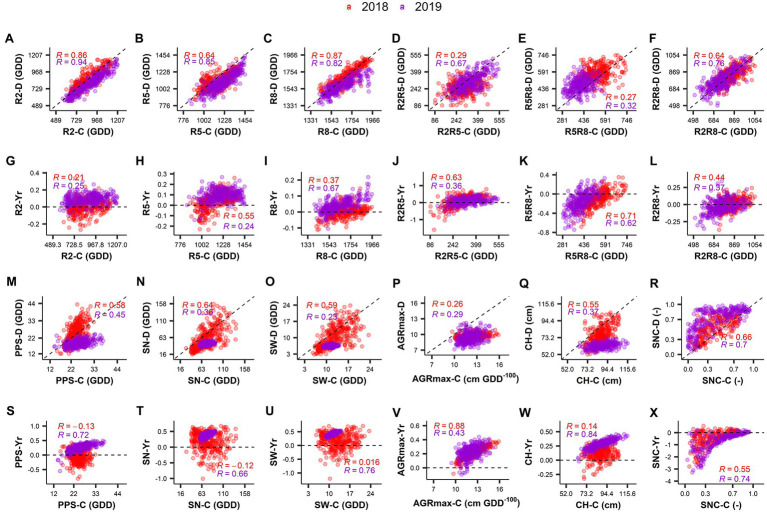
Correlation between BLUP values obtained in the control and drought treatments **(A–F**,**M–R)** and between drought index and control treatment **(G–L**,**S–X)** for the different traits in the 2 years investigated (2018 and 2019). “-**(C)**” and “-**(D)**” in the *Y*-axis labels represent the trait in control treatment and in drought treatment, respectively. “-Yr” in the labels of the *Y*-axis represents the drought index value for the respective trait. “R” value is the Pearson’s correlation coefficient. Different colors represent the year of the experiment. R2: Thermal time from sowing to full flowering; R5: Thermal time from sowing to beginning seed; R8: Thermal time from sowing to pod maturity; R2R5: Duration of pod formation; R5R8: Duration of seed development; R2R8: Thermal time from full flowering to pod maturity; PPS: Number of pods on the main stem; SN: Number of seeds per plant; SW: Seed weight per plant; Maximum absolute growth rate; CH: Maximum canopy height; SNC: Rate of senescence.

**Figure 5:**
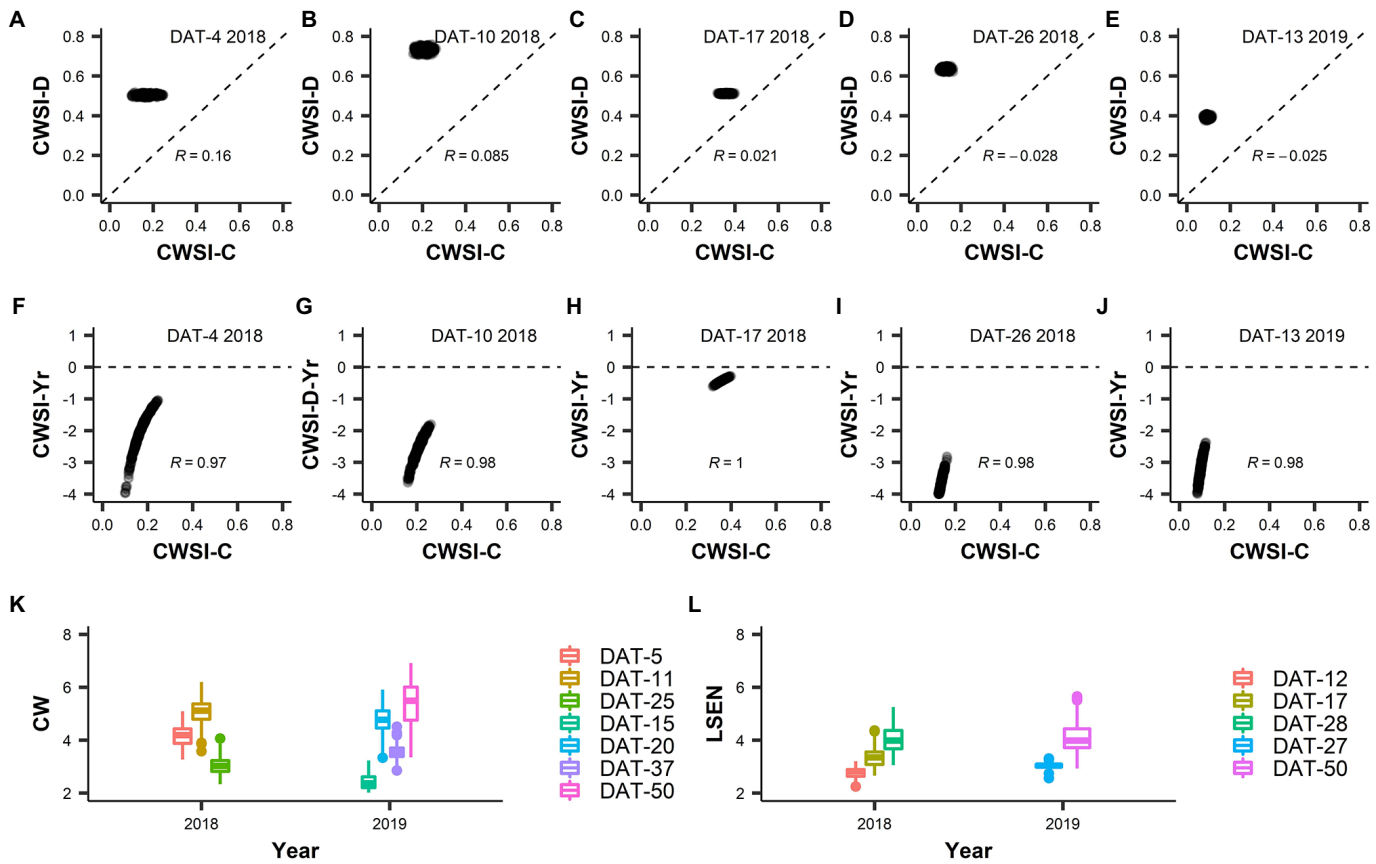
Crop Water Stress Index (CWSI), Canopy wilting (CW) and Leaf senescence (LSEN). **(A–E)** present the correlation of CWSI values between control and drought treatments. **(F–J)** present the correlation between CWSI drought index values (Yr) and values obtained in the control treatment for CWSI. In data labels, “DAT” is measurement day after drought treatment initiation, “2018” and “2019” represent the year of experiment and “R” value is the Pearson’s correlation coefficient. Legends in **(K,L)** represent the measurement days (DAT) for CW and LSEN, respectively.

**Figure 6:**
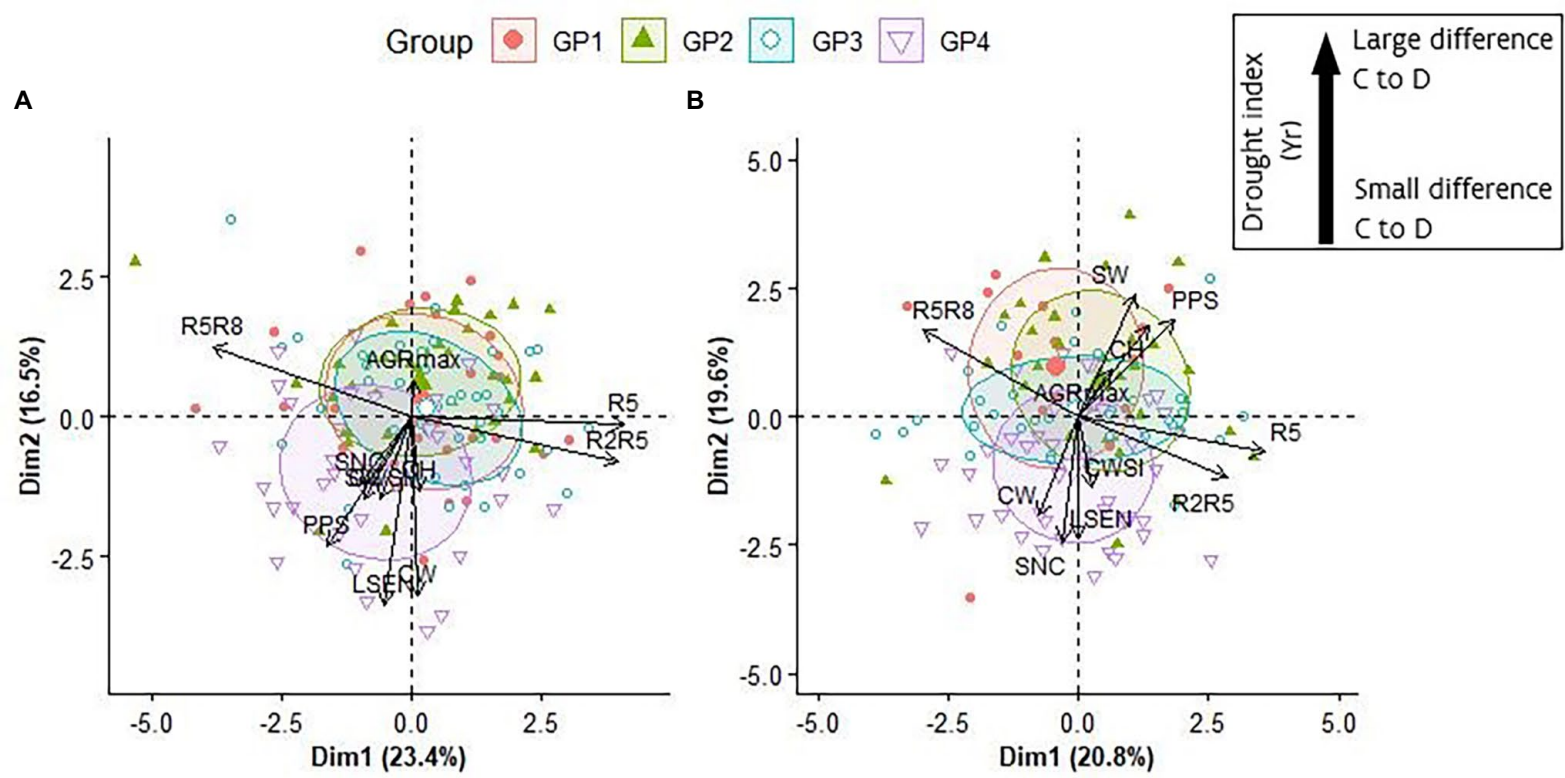
Representation of the first two axes of a Principal Component Analyses (PCA) performed on Yr data of R5, R2R5, R5R8, PPS, SW, AGRmax, CH, SNC and CWSI and BLUP values for LSEN and CW in 2018 **(A)** and in 2019 **(B)**. For representation purposes the “Yr” indicator has been removed from the variable names (e.g., in these plots “R5R8” represents “R5R8-Yr”). The inset in the figure does not apply to the interpretation of SNC values.

To quantify the impact of drought, we calculated the drought index (Yr; Equation 2) for the following traits: R2, R5, R8, R2R5, R2R8, R5R8, AGRmax, CH, SNC, PPS, SN, SW, and CWSI. We also included CW and LSEN, which had only been recorded in the drought field. As CC75 was achieved by most of the plots before the initiation of the drought treatment, it was not relevant to calculate Yr for this trait. Yr for DET was also not calculated as it is more related to the growth habit of accessions and not directly linked to a response to drought. First, we investigated the effect of drought on each trait based on Yr values (Tables 2 and 3; Figure 3) and the between-year correlation (Figure 4). We also related the response to drought (Yr) to the performance under well-watered conditions to check whether the performance of the accessions in the control field could explain their response to the drought treatment (Figures 4, 5). Finally, we performed a principal component analysis (PCA) to investigate the overall reaction of the accessions (Figure 6).

#### Traits Related to Plant Growth and Development

The CV of Yr values for R2, R5, R8, R2R5, R2R8, R5R8, AGRmax, CH, and SNC ranged from 18.5% to 43.6% (Table 2; Figure 3), suggesting a broad range of responses to drought. On average, the Yr values in 2019 were higher and more positive than in 2018 for R2, R5, R8, and CH, indicating accelerated development and shorter plants as a consequence of a long duration drought treatment in 2019 (drought reduced CH by 11 and 29% on average in response to short duration and long duration drought, respectively; Table 2; Figure 3K). A long drought treatment also induced much earlier senescence (negative Yr values) than a short drought treatment (26 and 110% higher SNC values in the drought fields in 2018 and 2019, respectively; Table 2 and Figure 3L). In both years the vast majority of Yr values for AGRmax was positive (Figure 3J) confirming that the drought treatment resulted in an overall reduction of the growth rate (average reduction of AGRmax of 22% in both years; Table 2), irrespective of the duration of drought treatment. These results indicate the accessions responded more strongly to the long drought treatment in 2019 as compared to the short duration drought in 2018, in terms of larger reduction of canopy height as well as accelerated development and senescence.

Strong correlations were found between control and drought fields for traits related to developmental stages (R2, R5, R8, R2R5, R5R8, and R2R8) in both years investigated (Figures 4A–F). This agrees with the high heritability calculated for these traits (see above). In 2018 (short duration drought treatment) no strong relationship was found between the Yr values and the control treatment values for R2 and R8 (Figures 4G,I). The accelerated development observed in 2019 as consequence of the long duration drought treatment (data are below the 1:1 line in Figure 4C), was stronger on the late maturing accessions (i.e., *R* = 0.67 between Yr values and values in control for R8, Figure 4I). Both of the short duration and the long duration drought treatments caused a prolongation of the duration of the seed development (R5R8) by 11–16% (Table 2; the majority of the dots are above the 1:1 line in Figure 4E). This was especially the case for many accessions of the early maturing GP4 (Supplementary Figure S7E). Conversely, the duration of pod formation (R2R5) was reduced by 9% in response of both of the short duration and the long duration drought treatments (Table 2).

In both years, drought caused a reduction of AGRmax and CH (Figures 4P,Q). After a long drought treatment, this reduction was stronger in the accessions that displayed the highest AGRmax values under well-watered conditions (Figure 4V, *R* = 0.43) or that grew taller under well-watered conditions (Figure 4W, *R* = 0.84). While SNC displayed a high variability, the variation was similar under drought and control treatments (Figure 4R). Drought accelerated senescence with a relatively stronger effect after a long drought treatment (Figure 4X), especially for many accessions of GP1 characterized by later senescence (Supplementary Figure S7L).

#### Physiological Traits

CWSI-Yr values were negative in all cases, indicating a reduction of the transpiration rate caused by drought in all accessions relative to their potential transpiration in the control (Figure 3M). In 2018 (short duration drought treatment) the largest difference between control and drought was recorded at DAT-26 (DAT: Days After Drought initiation), followed by DAT-10. This difference was small at DAT-17, indicating a lower level of stress in the drought field relative to DAT-10 and DAT-26. This was probably due to less severe environmental conditions at DAT-17 when the air relative humidity was higher (69% as compared to 41% at DAT-10, data not shown). However, the low level of variation of CWSI values prevents any further interpretation.

While LSEN increased as the drought treatment progressed, CW showed a more erratic behavior over time (Figures 5K,L). This was probably due to the way that CW was scored in this work. In comparison to LSEN, CW was more affected by environmental conditions (e.g., air temperature, humidity, solar radiation) when observations were made. In general, the correlation between LSEN values recorded at different dates was thus higher than the correlation between CW values recorded at different dates (Supplementary Figures S8, S9).

#### Traits Related to Yield

The response of seed yield-related traits to drought was stronger in 2019 (after a longer period of drought) than in 2018 (after a shorter period of drought), as indicated by a larger deviation of values from the diagonal in the comparisons of control vs. drought (Figures 4M–O). Interestingly, after a short drought treatment (2018), a substantial number of accessions displayed higher PPS values in the drought treatment compared to the control (as indicated by negative Yr values in Figure 4S). These accessions were mostly late maturing, belonging to GP1 and GP2 (Supplementary Figure S7G). In many cases this did not result in an increase of SN or SW (Figures 4T,U). Remarkably, the SN and SW values were very low in 2019 in both the drought and control fields, even though PPS in the control field of 2019 was comparable to that in 2018 (Figures 4M–O).

#### Multiple Trait Responses to Drought Stress

To get an overall view of the response to drought of this soybean collection, a PCA was performed using Yr data as well as CW and LSEN. For CW and LSEN, that were determined on different dates, the highest value observed for the accession at any date was considered in the analysis, and for CWSI, Yr data at the last measurement time was considered. As comparison of Yr values in 2018 (short duration drought stress) and 2019 (long duration drought stress) revealed a lack of correlation between years (Supplementary Figure S10), we carried out the analysis for each year separately. Preliminary data inspection for subsets of variables indicated that the following variables could be removed without loss of information: R2-Yr, R8-Yr, R2R8-Yr, and SN-Yr (Supplementary Figures S11, S12).

In general, a high level of consistency was found for the response of the accessions after a short and a long period of drought treatment, and the overall distribution of the GPs over the biplots (Figure 6). In both years, PC1 represented mainly the contrast between duration of pod development (R2R5-Yr) and duration of seed development (R5R8-Yr). The early maturing accessions of GP4 displayed the strongest responses for LSEN and canopy wilting (CW) after a short (2018) and long period (2019) of drought. The main difference between the biplots of short duration and long duration drought treatments is the length and direction of the arrows representing yield-related components (SW-Yr and PPS-Yr) and canopy height (CH-Yr). After a long period of drought (2019), a strong reduction in canopy height (mainly in late maturing accessions of GP1 and GP2) was associated with a strong reduction in yield-related traits. Remarkably, a strong response for CW and LSEN (mainly in early maturing accessions of GP4) was associated with less negative consequences for yield-related traits, as indicated by the opposite direction of arrows representing CW and LSEN on the one side, and SW-Yr and PSS-Yr on the other side (Figure 6B). This relationship is not apparent in the biplot of the short drought treatment (Figure 6A).

## Discussion

### A Collection With High Genetic and Phenotypic Diversity

Here we investigate the general characteristics and the response to drought of a large subset of the EUCLEG soybean collection described in Saleem et al. (2021). This collection is of relevance for breeding efforts in Europe and contains accessions from maturity classes I/II, 0, 00 and 000. For the purposes of this study, these were classified into four groups (GP1, GP2, GP3, and GP4). Phenotypic evaluation under well-watered conditions during 2 years revealed moderate to high levels of variation for most traits investigated. This was expected given the diverse origin of the accessions and the overall high level of genetic diversity within the collection (Saleem et al., 2021). A longer period of development of GP1 and GP2 accessions was observed which was expected as these accessions were comprised of relatively late maturing MGI/II and MG0 types. Conversely, accessions from GP3 and GP4, displayed a shorter period of development and relatively faster growth. As discussed by Aper et al. (2016), in the location where this study was conducted, the strong vegetative development of early maturing accessions confers them good weed suppression capabilities, and enables the accumulation of the sufficient photosynthates for flowering and seed filling. A decreasing trend for yield traits (PPS, SN, and SW) was observed over maturity duration, with larger values in GP1 and GP2 and smaller values in GP3 and GP4. A similar trend was also described by Aper et al. (2016). Remarkably, the inter-year differences for yield traits (SN and SW) was particularly high for GP1 and GP2 accessions. These accessions originate mostly from Eastern and Southern Europe, while GP3 and GP4 accessions originate mostly from Western and Northern Europe (Supplementary Table S1). Given the narrow range of adaptation of soybean varieties bred for a specific region due to sensitivity to photoperiod and temperature (Song et al., 2019), the more stable yield observed in this study for GP3 and GP4 can be explained by a better adaptation of these accessions to Northwest European conditions.

We observed also a wide range of variation for phenological traits (R2, R5, and R8) and a decreasing trend in duration of developmental phases from GP1 to GP4. Surprisingly there was no clear relationship between yield-related traits and either duration of pod formation (R2R5) or seed development (R5R8). The thermal time from sowing to pod maturity (R8) was also not correlated with R2R5 or R5R8. A long duration of reproductive development has been proposed as a strategy to improve soybean yield without affecting the total length of the growing cycle (Metz et al., 1985; Cooper, 2003), and a simulation study predicted a positive relationship between yield and the thermal time to flowering and pod maturity in Northern Europe (Boulch et al., 2021). Our results indicate that the EUCLEG collection contains accessions with the required combination of a long duration of reproductive development and high values of yield traits, with possibly no direct impact on the total length of the growing period.

It has been reported that semi-determinate soybean genotypes can compensate for short adverse periods because of their capacity to produce reproductive organs for longer than determinate types (Zhang et al., 2019a). Semi-determinacy is also considered a good characteristic to introduce in early maturing soybean varieties, provided that taller plants do not have increased risk of lodging (Kato et al., 2019). Correspondingly, multiple studies have expressed the need to develop semi-determinate early maturity soybean material in Europe (Rosenzweig et al., 2003; Schori et al., 2003; Aper et al., 2016). We have found a high range of variation for indeterminacy (DET) in the EUCLEG collection, but we also found that late maturing types (GP1) are on average more indeterminate than early maturing ones (GP4). However, DET correlated positively with the length of the period between flowering and maturity (R2R8) in GP3 and GP4 (*R* = 0.5–0.6, data not shown), suggesting that by using this collection, semi-determinacy could be combined with a long duration of reproductive development in early maturing types.

The high heritability of traits related to phenological development including R2, R5, and R8 was according to expectations (Zhang et al., 2015; Li et al., 2019). A variable performance for yield-related traits as noticed in our study in the control fields was also expected given the complex quantitative nature of yield, with a strong influence of the environment (Kuswantoro, 2019; Xavier and Rainey, 2020). In contrast, canopy height (CH) and number of pods on the main stem (PPS) were relatively more stable across 2 years under well-watered conditions (*R* = 0.55 and 0.48, respectively). A significant positive correlation between these traits highlights their significance to improve yield in soybean.

### Anticipated Multiple and Diverse Responses to Drought in Soybean

Given the high level of diversity contained in the EUCLEG collection, we anticipated that multiple and diverse responses to drought would be present among the genotypes investigated. We therefore performed a thorough evaluation of multiple traits. Traits that are directly related to drought response including LSEN, canopy wilting (CW) and canopy temperature (CWSI), were combined with traits that describe the growth (AGRmax, CH), the developmental path (R2, R5, and R8) and the duration of reproductive development phases (R2R5, R5R8, and R2R8). In addition, because drought resistance should not compromise productivity, yield-related parameters (PPS, SN, and SW) were also considered in the evaluation.

We observed a low to medium level of variation for canopy wilting (CW) and LSEN (Table 3). In general, the variation increased as the treatment progressed, indicating a differential response of the accessions to drought. Multiple mechanisms have been proposed to result in slow canopy wilting including low stomatal conductance, deep rooting, constant transpiration under high vapor pressure deficit and low radiation use efficiency (Kunert and Vorster, 2020). While slow or delayed wilting is considered useful in soybean because it can protect yield under drought conditions (Ye et al., 2020), it was not easy to evaluate wilting symptoms in our experiment. This was probably due to the influence of the environmental conditions prevalent at the time of evaluation. Nonetheless, we found a significant correlation between CW and LSEN. As the evaluation of LSEN seems to be less prone to the particular environmental conditions at the time of the evaluation, we consider this a more robust indicator of the response of soybean to water deficit. We did not find a clear relationship between LSEN and drought response for yield-related traits after a short period of drought, but accessions of GP4 displayed higher LSEN values on average than those of other GPs. After a long period of drought, GP4 accessions displayed higher LSEN values on average and were relatively less affected for yield-related traits (determined as number of pods on the main stem and seed weight per plant) than those of other GPs. As GP4 accessions generally grow faster and mature earlier, it is possible that these characteristics help them to show a less reduction in yield when the drought condition is maintained for long. Anyhow our results suggest that under long drought stress, the stronger signs of LSEN might be associated with a high resistance, at least among early maturing accessions. As the level of variation for LSEN within GP4 is substantial, a further study especially in early maturing accessions may help to clarify the relationship between LSEN and response for yield under drought conditions.

Based on a comparison of different indices and approaches, De Swaef et al. (2021) concluded that CWSI can be a complementary criterion to detect differential responses to drought stress in perennial grasses. CWSI has also been employed to determine the level of stress and to schedule irrigation in soybean (Candogan et al., 2013; Tekelioğlu et al., 2017), and Anda et al. (2019) reported higher CWSI values in soybean under drought as compared to a control treatment. Our results were in accordance with this, as higher CWSI values were noted for the drought treatments than well-watered conditions. However, the variation for CWSI in the drought treatments was low. As soybean is a rather isohydric species, plants probably close their stomata even when they are exposed to moderate drought stress (Tardieu and Simonneau, 1998), what might explain the lack of variation observed.

In soybean, a longer period of grain filling has been shown to be advantageous for yield potential at high latitudes (Cooper, 2003). A simulation study estimated an optimum grain filling duration of 60 days for soybean in Northern France (Boulch et al., 2021). In our experiments, drought lengthened the duration of seed development (R5R8, corresponding to the seed filling duration), which was due to an earlier shift to R5 (start of seed formation) which led a shortening of R2R5 (corresponding to the period during which new flowers and pods are formed). Drought response for pod formation duration (R2R5) or grain filling duration (R5R8) was independent from drought response for yield-related traits, as illustrated by the PCA analysis. The reduction of seed yield-related traits under the short duration drought was less prominent in later maturing accessions of GP1 and GP2. The late maturing accessions of GP1 and GP2 in the EUCLEG collection also displayed a higher degree of indeterminacy. It is possible that the drought treatment caused the cessation of flower and pod formation in these accessions, but as they are also more indeterminate, they might have produced more flowers and pods after the end of the drought treatment with less yield penalty than accessions from other maturity groups. As accessions of GP4 are more determinate, it is possible that only the flowers and pods that had been formed before the drought treatment were able to produce seeds, causing a larger difference for yield-related traits between control and drought fields.

Also after a long period of drought, the response for pod formation duration (R2R5) or grain filling duration (R5R8) was independent from drought response for yield-related traits, as illustrated by the PCA analysis in 2019. The prominent responses to a long drought treatment in late maturing accessions of GP1 and GP2 were a reduction in maximum growth rate (AGRmax) and canopy height (CH) along with a reduction in yield-related traits (PPS and SW). Contrary to what was observed after a short period of drought in 2018, the indeterminate behavior of accessions of GP1 and GP2 was insufficient to protect the yield after a longer drought treatment in 2019. This is probably a reflection of irreversible damage that is common under severe stress (Sehgal et al., 2017; Wei et al., 2018).

### Replication of a Drought Experiment in the Field Proved Difficult

Rain-out shelters such as those used for this study allow a good evaluation of the response of plants to drought in the field, as they have limited impact on the air temperature or light conditions and root growth is not limited by the size of the plot as in greenhouse or growth chamber experiments (De Swaef et al., 2021). Using moisture sensors, we also succeeded in attaining similar soil conditions over the 2 years under study. When the rain-out shelters were first positioned over the plots, the average soil moisture content was similar (0.11 v/v in 2018 and 0.12 v/v in 2019), and it dropped to similar levels during the treatment (0.06 v/v and 0.05 v/v in 2018 and 2019, respectively). However, other environmental parameters that strongly affect soybean phenology and development (Wang et al., 1997; Salem et al., 2007; Alsajri et al., 2019; Kumagai and Takahashi, 2020) are difficult to manipulate under field conditions, making it impossible to completely eliminate year-to-year variability. In 2019 the lower air temperature, lower solar radiation and higher relative humidity retarded the development of stress symptoms as compared to 2018. This, in combination with our decision to maintain the drought treatment for a longer period in 2019 than in 2018, had important consequences for the performance of the plants. This led to different environmental conditions experienced by the plants in 2018 and 2019. Correspondingly, we found low to medium stability for most of the trait responses across the 2 years, illustrating that plant responses in a specific drought scenario are not only affected by the reduced soil moisture level but also by other environmental components including temperature, solar radiation and vapor pressure deficit (Tardieu, 2012).

One way to replicate the drought treatment would be to screen for drought resistance using only years with similar environmental characteristics, but this is not practical and perhaps not even possible. Another approach can be to choose areas associated with stable environment across years (Saryoko et al., 2017). An alternative approach, as discussed by Tardieu (2012), is the combination of different methods including phenotyping and modeling: phenotyping in controlled conditions to identify parameters of models, simulation of trait values in a large range of climatic scenarios by using a model with genotype-specific parameters and, finally, testing these models in a limited number of field experiments. This explicitly takes into account the year-to-year variability of drought scenarios, and can be combined with model assisted breeding.

## Conclusion

We found a wide range of phenotypic diversity for absolute growth rate, canopy height, degree of indeterminacy, phenology and yield-related traits under well-watered conditions in a diverse collection of soybean accessions of relevance for breeding in Europe. Drought applied at the reproductive stage in two seasons brought about diverse responses in this collection. The long duration drought treatment (for 6–7 weeks) in 2019 caused a much stronger response as compared to the short duration drought (for 3–4 weeks) in 2018. Main responses were an average reduction of 11–29% in maximum canopy height, an average reduction of 22% in maximum absolute growth rate and an acceleration of the rate of senescence by 26–110%. Drought also caused a reduction of 9% in the duration of pod formation but conversely an increase of 11–16% in the duration of seed development.

The characteristics of the drought treatment in 2018 and 2019 were different, which resulted in differential responses of the accessions over the 2 years. When a short period of drought was applied (2018) the earlier cessation of flower and pod formation allowed a less pronounced reduction of yield-related traits. A longer duration of the drought stress treatment (2019) brought about a different response. Under these conditions (long drought stress treatment), the accessions that displayed a strong reduction in canopy height (cessation of growth) were also the most affected for yield-related traits. These results suggest that under the conditions associated with a short period of drought stress, drought resistance criteria can be based on yield-related traits, while resistance to long drought stress can be improved by selecting for genotypes that are able to maintain growth. Although stronger signs of LSEN and canopy wilting helped some accessions (mainly GP4) to protect their yield (determined as number of pods on the main stem and seed weight per plant) under long drought stress, further exploration of this relationship especially in early maturing accessions is necessary.

## Data Availability Statement

The original contributions presented in the study are included in the article/Supplementary Material, further inquiries can be directed to the corresponding author.

## Author Contributions

AS, JA, and IR-R conceived the study. AS and JA performed the research. PL and IB-S organized the UAV flights and pre-processed the data. AS, HM, and PQ analyzed the data. AS and IR-R drafted the manuscript. AS, JA, HM, IB-S, PQ, PL, TS, and IR-R interpreted the results and elaborated the manuscript. All authors read and approved the final version of the manuscript.

## Funding

This project has received funding from the European Union’s Horizon 2020 Program for Research & Innovation under grant agreement no. 727312 (project: “EUCLEG – Breeding forage and grain legumes to increase EU’s and China’s protein self-sufficiency”).

## Conflict of Interest

The authors declare that the research was conducted in the absence of any commercial or financial relationships that could be construed as a potential conflict of interest.

## Publisher’s Note

All claims expressed in this article are solely those of the authors and do not necessarily represent those of their affiliated organizations, or those of the publisher, the editors and the reviewers. Any product that may be evaluated in this article, or claim that may be made by its manufacturer, is not guaranteed or endorsed by the publisher.

## Footnotes

^1^ http://www.qgis.org/
