# Supplementary material for: Response of a Diverse European Soybean Collection to “Short Duration” and “Long Duration” Drought Stress
Source: Front Plant Sci. 2022 Feb 17;13:818766. doi: 10.3389/fpls.2022.818766 (PMC8891225; doi:10.3389/fpls.2022.818766)
Supplement: Supplementary file 1 [file Data_Sheet_1.docx]

Supplementary Material


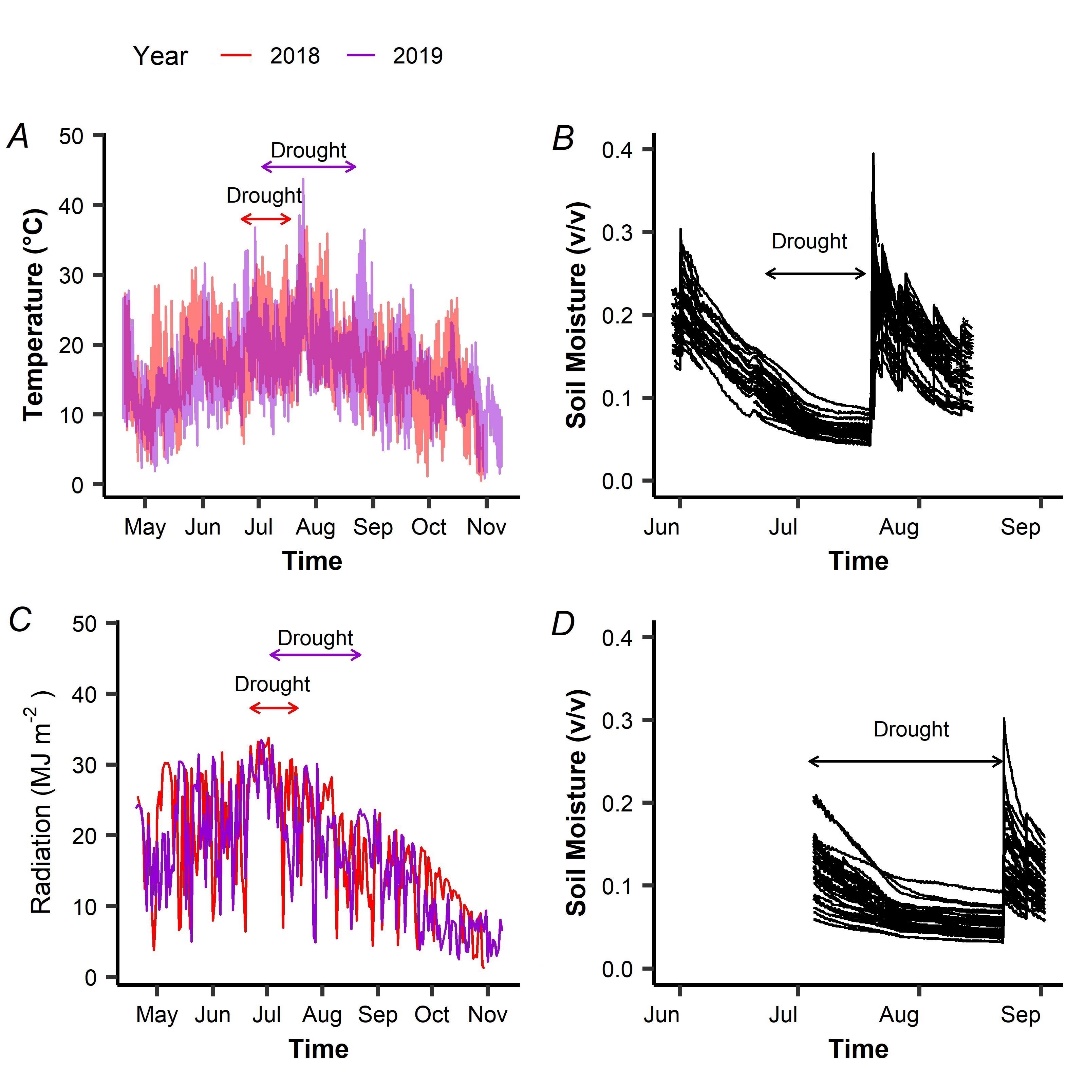


**Figure S1.** Environmental conditions in 2018 and 2019. (A) Daily minimum and maximum temperature, (B) Sum of daily solar shortwave radiation, (C) Evolution of the soil moisture content in the drought field during 2018, and (D) Evolution of the soil moisture content in the drought field during 2019. The data in C and D were obtained using 36 TDR sensors. The arrows delineate the period during which the rain-out shelters were covering the plants of the drought field.


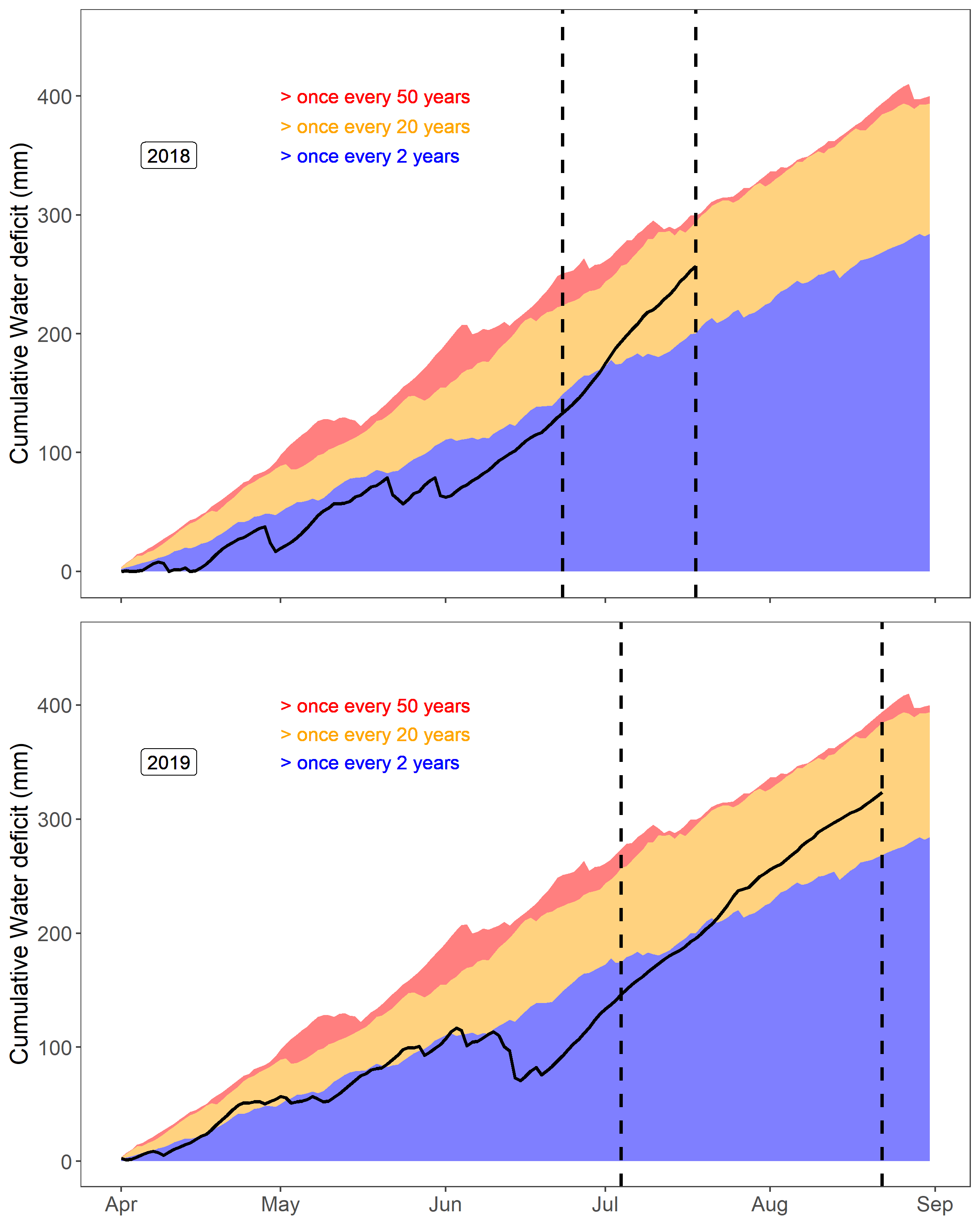


**Figure S2.** Cumulative water deficit (CWD) in the drought fields in 2018 and 2019. CWD was calculated as the accumulation of the difference between daily reference evapotranspiration (ET0 in mm) and precipitation (P in mm), starting at 1 April. Colored sections represent the long-term CWD statistics for the location in which the field trials were performed. These values were calculated using combined weather data sets (from 1979 to 2021) from the Joint Research Centre (JRC MARS Meteorological Database) and the Royal Meteorological Institute (KMI). The vertical lines delineate the period during which the rain-out shelters were covering the plants of the drought fields.


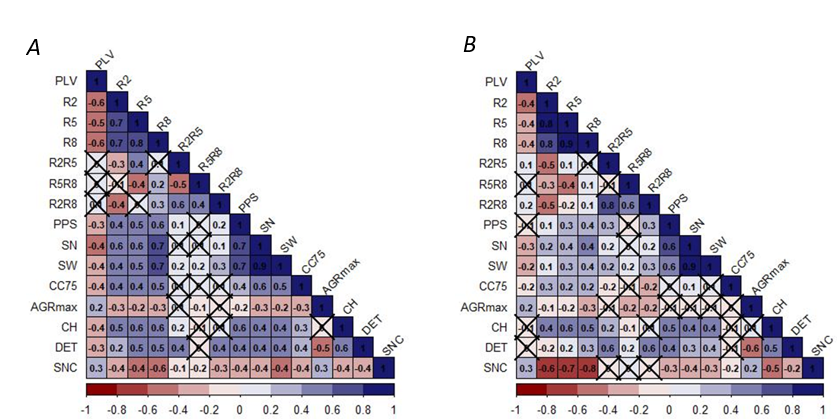


**Figure S3.** Correlation between traits measured under optimal conditions (control treatment) in 2018 (A) and in 2019 (B). PLV: Plant length up to the second node; R2: Thermal time from sowing to full flowering; R5: Thermal time from sowing to beginning seed; R8: Thermal time from sowing to pod maturity; R2R5: Duration of pod formation; R5R8: Duration of seed development; R2R8: Thermal time from full flowering to pod maturity; PPS: Number of pods per  main stem; SN: Number of seeds per plant; SW: Seed weight per plant; CC75: Thermal time to canopy cover 75%; AGRmax: Maximum absolute growth rate; CH: Maximum canopy height; DET: Degree of indeterminacy; SNC: Rate of senescence.

*
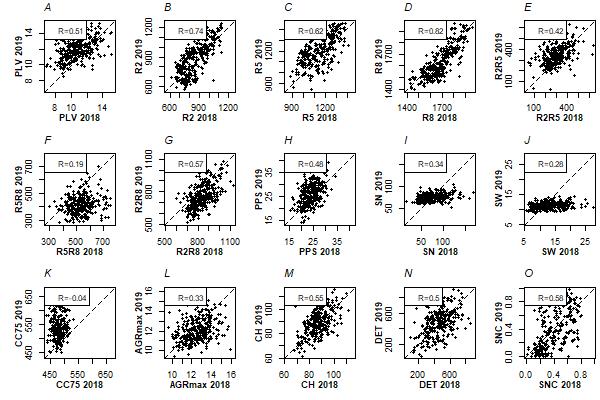
*

**Figure S4.** Correlation of BLUP values between 2018 and 2019 from the control treatment. PLV: Plant length up to the second node; R2: Thermal time from sowing to full flowering; R5: Thermal time from sowing to beginning seed; R8: Thermal time from sowing to pod maturity; R2R5: Duration of pod formation; R5R8: Duration of seed development; R2R8: Thermal time from full flowering to pod maturity; PPS: Number of pods per  main stem; SN: Number of seeds per plant; SW: Seed weight per plant; CC75: Thermal time to canopy cover 75%; AGRmax: Maximum absolute growth rate; CH: Maximum canopy height; DET: Degree of indeterminacy; SNC: Rate of senescence.


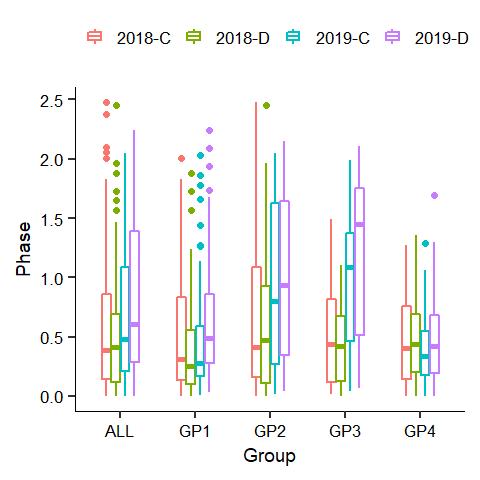


**Figure S5.** Developmental phase of the plants in the control and drought fields at the time that the drought treatment was initiated. ‘Labels on the X-axis represent the four groups of accessions with different sowing moments (for details see materials and methods)., and ‘ALL’ represents the values for all accessions together. The value on the Y-axis is the score of the development phase determined from the sigmoid function as described in materials and methods, where, 0: vegetative phase, 0-1: start of flowering (R1), 1-2: full flowering (R2) and 2-3: beginning pod (R3). ‘-C’ and ‘-D’ in legends represent the control and the drought treatments respectively.


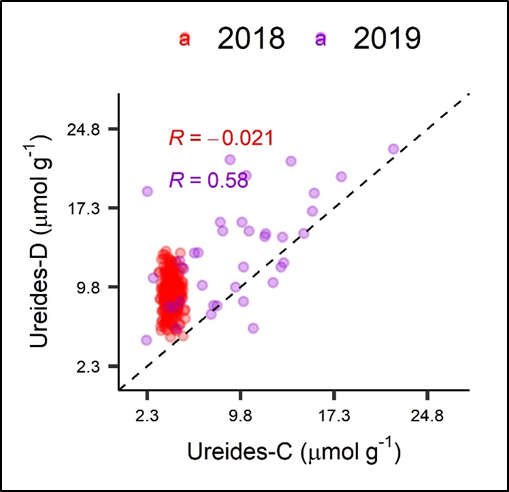


**Figure S6.** Stem ureides content of complete set of accessions in 2018 and of a subset of 40 accessions in 2019. ‘-C’ and ‘-D’ in the axis labels represent the control treatment and drought treatment respectively.

*
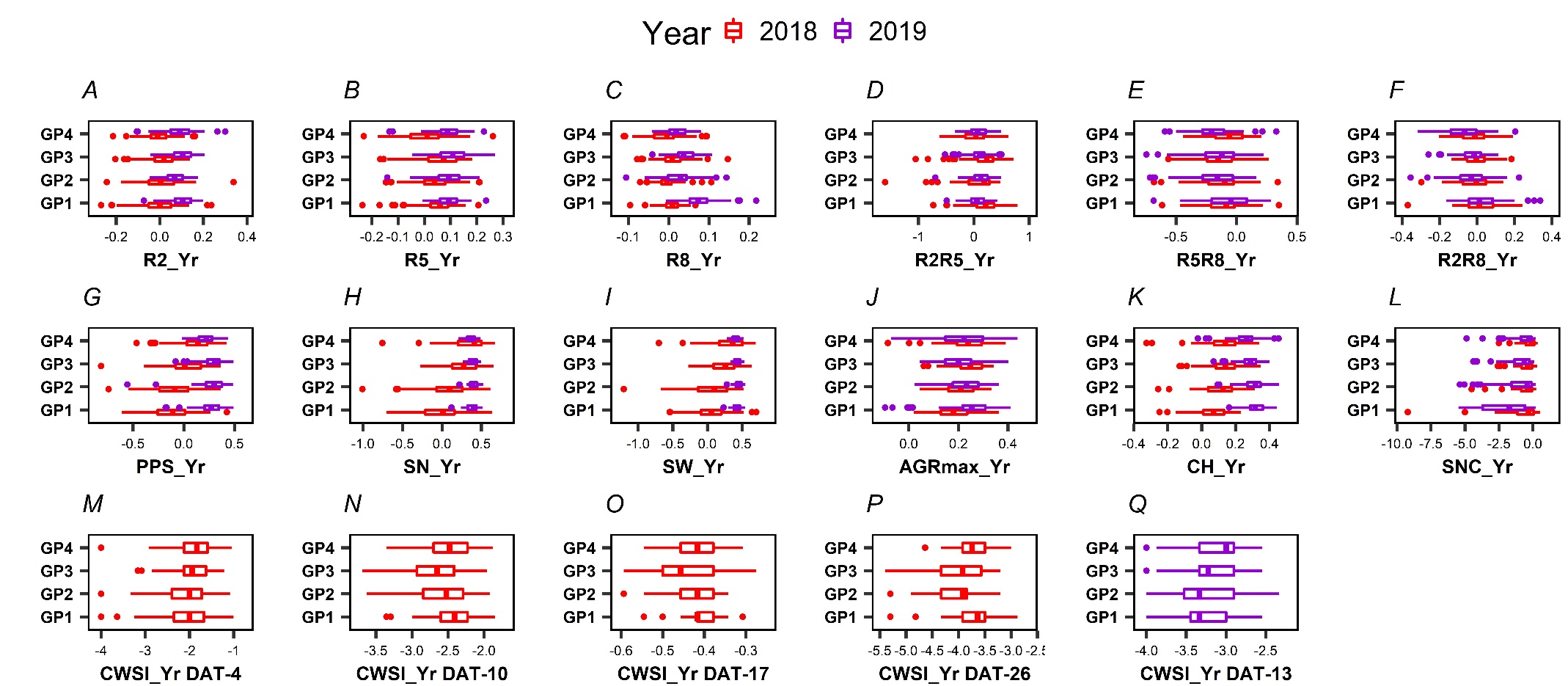
*

**Figure S7.** Drought index values (Yr) for the different traits obtained for the four groups of accessions in which the collection was divided. 2018 corresponds to a drought treatment of short duration; 2019 corresponds to a drought treatment of long duration. In X-axis labels, ‘Yr’ is the drought index and ‘DAT’ is measurement day after drought treatment initiation. R2: Thermal time from sowing to full flowering; R5: Thermal time from sowing to beginning seed; R8: Thermal time from sowing to pod maturity; R2R5: Duration of pod formation; R5R8: Duration of seed development; R2R8: Thermal time from full flowering to pod maturity; PPS: Number of pods per  main stem; SN: Number of seeds per plant; SW: Seed weight per plant; CC75: Thermal time to canopy cover 75%; AGRmax: Maximum absolute growth rate; CH: Maximum canopy height; DET: Degree of indeterminacy; SNC: Rate of senescence; CWSI: Crop water stress index.


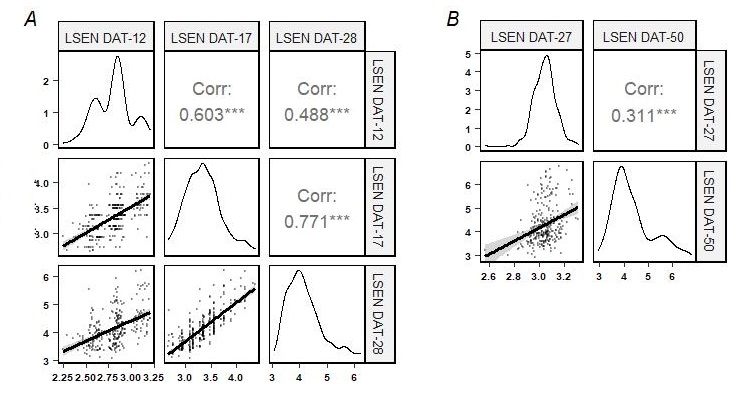


**Figure S8.** Correlation between LSEN (Leaf senescence) values determined at different dates after initiation of the drought treatment (DAT) in 2018 (A) and in 2019 (B). Corresponding Pearson’s correlation coefficient values are given above the diagonal.


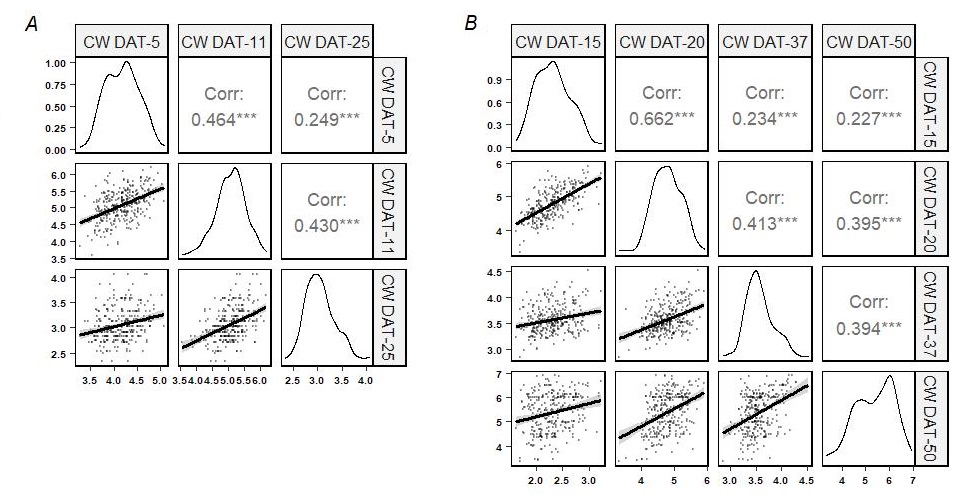


**Figure S9.** Correlations between CW (Canopy wilting) values determined at different dates after initiation of the drought treatment (DAT) in 2018 (A) and in 2019 (B). Corresponding Pearson’s correlation coefficient values are given above the diagonal.

*
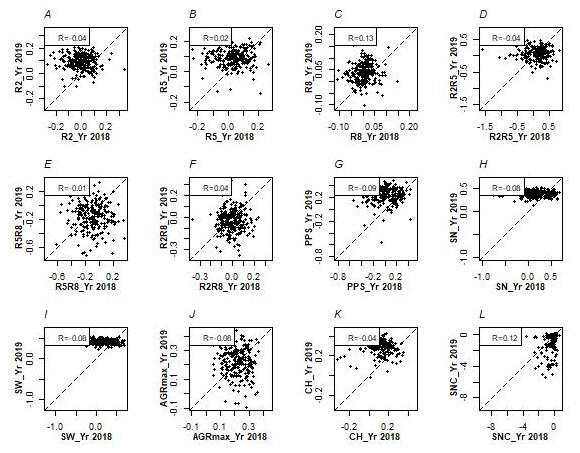
*

**Figure S10.** Correlation between drought index values (Yr) obtained for the different traits in 2018 and 2019. R2: Thermal time from sowing to full flowering; R5: Thermal time from sowing to beginning seed; R8: Thermal time from sowing to pod maturity; R2R5: Duration of pod formation; R5R8: Duration of seed development; R2R8: Thermal time from full flowering to pod maturity; PPS: Number of pods per  main stem; SN: Number of seeds per plant; SW: Seed weight per plant; CC75: Thermal time to canopy cover 75%; AGRmax: Maximum absolute growth rate; CH: Maximum canopy height; DET: Degree of indeterminacy; SNC: Rate of senescence.


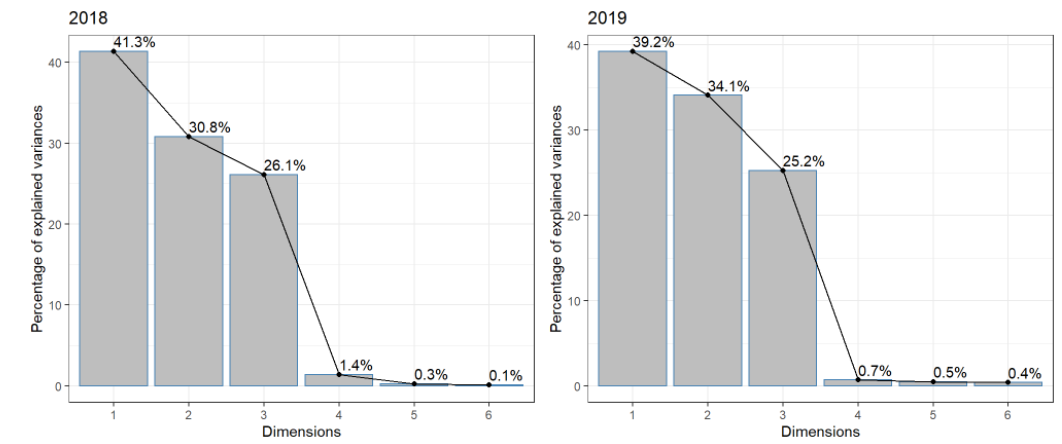


**Figure S11.** Percentage of variance explained by the first six principal components of a PCA in which only the following variables were included: Thermal time from sowing to full flowering (R2); Thermal time from sowing to beginning seed (R5); Thermal time from sowing to pod maturity (R8); Duration of pod formation (R2R5): Duration of seed development (R5R8); Thermal time from full flowering to pod maturity (R2R8). Left: 2018 (short duration drought stress); Right: 2019 (long duration drought stress).


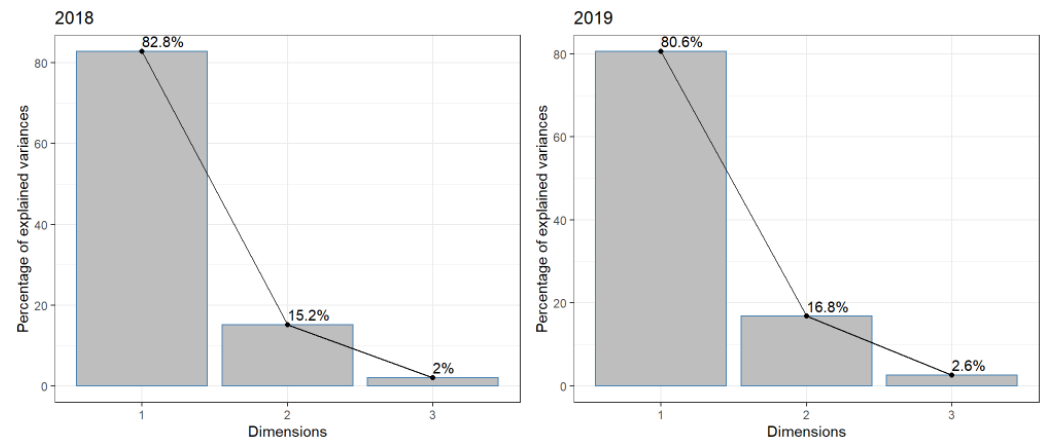


**Figure S12.** Percentage of variance explained by the first six principal components of a PCA in which only the following variables were included: Number of pods per  main stem (PPS); Number of seeds per plant (SN); Seed weight per plant (SW). Left: 2018 (short duration drought stress); Right: 2019 (long duration drought stress).

**Table S1.** Description of the accessions included in this study. ‘Flowering time’ and ‘Maturity time’ is the cumulated thermal time in growing degree days (GDD) from sowing, average of two years (2018 and 2019) from the control treatment of the current study.

| Accession ID | Geographical origin | Country | Source of Accessions* | Accession type | Growth group | Number of replications | Flowering time | Maturity time |
| --- | --- | --- | --- | --- | --- | --- | --- | --- |
| EUC_GM_001 | Southern Europe | Serbia | IFVCNS | Variety | GP1 | 9 | 984 | 1908 |
| EUC_GM_002 | China | China | USDA-ARS | Variety | GP1 | 6 | 993 | 1943 |
| EUC_GM_003 | China | China | USDA-ARS | Variety | GP1 | 6 | 896 | 1812 |
| EUC_GM_004 | USA | USA | USDA-ARS | Variety | GP1 | 3 | 832 | 1839 |
| EUC_GM_005 | Japan | Japan | USDA-ARS | Variety | GP1 | 3 | 1015 | 1863 |
| EUC_GM_006 | Southern Europe | Serbia | IFVCNS | Variety | GP1 | 3 | 996 | 1921 |
| EUC_GM_007 | Southern Europe | Italy | ERSA | Variety | GP1 | 1 | 971 | 1931 |
| EUC_GM_008 | Southern Europe | Italy | ERSA | Variety | GP1 | 1 | 854 | 1893 |
| EUC_GM_009 | Canada | Canada | IFVCNS | Variety | GP1 | 1 | 631 | 1622 |
| EUC_GM_010 | Southern Europe | Italy | ERSA | Variety | GP1 | 1 | 902 | 1872 |
| EUC_GM_011 | Southern Europe | Italy | ERSA | Variety | GP1 | 1 | 816 | 1891 |
| EUC_GM_012 | Eastern Europe | Russia | USDA-ARS | Breeding line | GP1 | 1 | 903 | 1766 |
| EUC_GM_013 | Southern Europe | Italy | ERSA | Variety | GP1 | 1 | 1002 | 1842 |
| EUC_GM_014 | Eastern Europe | Romania | NARDI Fundulea | Variety | GP1 | 1 | 935 | 1935 |
| EUC_GM_015 | Eastern Europe | Romania | SCDA Turda | Variety | GP1 | 1 | 962 | 1923 |
| EUC_GM_016 | Eastern Europe | Ukraine | IFRAP NAAS | Variety | GP1 | 1 | 685 | 1698 |
| EUC_GM_017 | Eastern Europe | Russia | USDA-ARS | Breeding line | GP1 | 1 | 851 | 1744 |
| EUC_GM_018 | Eastern Europe | Romania | SCDA Turda | Variety | GP1 | 1 | 954 | 1885 |
| EUC_GM_019 | Southern Europe | Italy | ERSA | Variety | GP1 | 1 | 680 | 1740 |
| EUC_GM_020 | Canada | Canada | ART | Variety | GP1 | 1 | 525 | 1786 |
| EUC_GM_021 | Canada | Canada | University of Guelph | Variety | GP1 | 1 | 811 | 1876 |
| EUC_GM_022 | Eastern Europe | Romania | NARDI Fundulea | Variety | GP1 | 1 | 822 | 1840 |
| EUC_GM_023 | Southern Europe | Italy | ERSA | Variety | GP1 | 1 | 1006 | 1899 |
| EUC_GM_024 | USA | USA | IFVCNS | Variety | GP1 | 1 | 794 | 1797 |
| EUC_GM_025 | Eastern Europe | Bulgaria | IFC-Pleven | Variety | GP1 | 1 | 884 | 1886 |
| EUC_GM_026 | Eastern Europe | Bulgaria | IFC-Pleven | Variety | GP1 | 1 | 958 | 1917 |
| EUC_GM_027 | Eastern Europe | Bulgaria | IFC-Pleven | Variety | GP1 | 1 | 976 | 1841 |
| EUC_GM_028 | Southern Europe | Italy | ERSA | Variety | GP1 | 1 | 644 | 1782 |
| EUC_GM_029 | Southern Europe | Serbia | MRIZP | Variety | GP1 | 1 | 966 | 1947 |
| EUC_GM_030 | Southern Europe | Serbia | MRIZP | Variety | GP1 | 1 | 930 | 1921 |
| EUC_GM_031 | China | China | USDA-ARS | Landrace | GP1 | 1 | 660 | 1778 |
| EUC_GM_032 | China | China | USDA-ARS | Landrace | GP1 | 1 | 806 | 1722 |
| EUC_GM_033 | China | China | USDA-ARS | Landrace | GP1 | 1 | 759 | 1772 |
| EUC_GM_034 | Eastern Europe | Georgia | USDA-ARS | Breeding line | GP1 | 1 | 806 | 1826 |
| EUC_GM_035 | China | China | USDA-ARS | Variety | GP1 | 1 | 706 | 1800 |
| EUC_GM_036 | Eastern Europe | Russia | USDA-ARS | Breeding line | GP1 | 1 | 696 | 1730 |
| EUC_GM_037 | China | China | USDA-ARS | Landrace | GP1 | 1 | 989 | 1874 |
| EUC_GM_038 | Southern Europe | Italy | ERSA | Variety | GP1 | 1 | 703 | 1868 |
| EUC_GM_039 | Southern Europe | Italy | ERSA | Variety | GP1 | 1 | 849 | 1907 |
| EUC_GM_040 | Eastern Europe | Romania | IPK | Variety | GP1 | 1 | 755 | 1733 |
| EUC_GM_041 | Southern Europe | Italy | ERSA | Variety | GP1 | 1 | 830 | 1894 |
| EUC_GM_042 | Eastern Europe | Hungary | IPK | Landrace | GP1 | 1 | 823 | 1843 |
| EUC_GM_043 | Western Europe | Germany | IPK | Variety | GP1 | 1 | 557 | 1841 |
| EUC_GM_044 | China | China | USDA-ARS | Variety | GP1 | 1 | 907 | 1871 |
| EUC_GM_045 | Southern Europe | Italy | ERSA | Variety | GP1 | 1 | 935 | 1922 |
| EUC_GM_046 | Western Europe | Germany | IPK | Variety | GP1 | 1 | 1031 | 1981 |
| EUC_GM_047 | China | China | USDA-ARS | Landrace | GP1 | 1 | 984 | 1909 |
| EUC_GM_048 | USA | USA | USDA-ARS | Variety | GP1 | 1 | 945 | 1763 |
| EUC_GM_049 | Eastern Europe | Romania | NARDI Fundulea | Variety | GP1 | 1 | 923 | 1852 |
| EUC_GM_050 | Eastern Europe | Romania | SCDA Turda | Variety | GP1 | 1 | 937 | 1911 |
| EUC_GM_051 | USA | USA | USDA-ARS | Variety | GP1 | 1 | 957 | 1779 |
| EUC_GM_052 | Western Europe | Germany | USDA-ARS | Landrace | GP1 | 1 | 890 | 1721 |
| EUC_GM_053 | Eastern Europe | Ukraine | USDA-ARS | Landrace | GP1 | 1 | 846 | 1732 |
| EUC_GM_054 | China | China | USDA-ARS | Variety | GP1 | 1 | 616 | 1720 |
| EUC_GM_055 | Eastern Europe | Romania | SCDA Turda | Variety | GP1 | 1 | 670 | 1864 |
| EUC_GM_056 | Eastern Europe | Romania | SCDA Turda | Variety | GP1 | 1 | 770 | 1866 |
| EUC_GM_057 | China | China | USDA-ARS | Landrace | GP1 | 1 | 523 | 1740 |
| EUC_GM_058 | USA | USA | IFVCNS | Variety | GP1 | 1 | 756 | 1739 |
| EUC_GM_059 | China | China | USDA-ARS | Variety | GP1 | 1 | 767 | 1857 |
| EUC_GM_060 | China | China | USDA-ARS | Variety | GP1 | 1 | 594 | 1765 |
| EUC_GM_061 | China | China | USDA-ARS | Variety | GP1 | 1 | 771 | 1853 |
| EUC_GM_062 | China | China | USDA-ARS | Variety | GP1 | 1 | 849 | 1879 |
| EUC_GM_063 | China | China | USDA-ARS | Variety | GP1 | 1 | 736 | 1886 |
| EUC_GM_064 | China | China | USDA-ARS | Variety | GP1 | 1 | 938 | 1837 |
| EUC_GM_065 | Japan | Japan | USDA-ARS | Variety | GP1 | 1 | 768 | 1775 |
| EUC_GM_066 | Eastern Europe | Ukraine | IPK | Variety | GP1 | 1 | 770 | 1771 |
| EUC_GM_067 | Western Europe | Germany | IPK | Variety | GP1 | 1 | 877 | 1822 |
| EUC_GM_068 | USA | USA | USDA-ARS | Variety | GP1 | 1 | 734 | 1766 |
| EUC_GM_069 | Western Europe | France | IPK | Landrace | GP1 | 1 | 820 | 1723 |
| EUC_GM_070 | Western Europe | Germany | IPK | Landrace | GP1 | 1 | 707 | 1754 |
| EUC_GM_071 | Southern Europe | Serbia | IFVCNS | Breeding line | GP1 | 1 | 835 | 1862 |
| EUC_GM_072 | Eastern Europe | Romania | SCDA Turda | Variety | GP1 | 1 | 855 | 1859 |
| EUC_GM_073 | USA | USA | USDA-ARS | Variety | GP1 | 1 | 757 | 1704 |
| EUC_GM_074 | Unknown | Unknown | IPK | Variety | GP1 | 1 | 710 | 1751 |
| EUC_GM_075 | Southern Europe | Italy | ERSA | Variety | GP1 | 1 | 835 | 1902 |
| EUC_GM_076 | Western Europe | Germany | IFVCNS | Variety | GP1 | 1 | 766 | 1738 |
| EUC_GM_077 | Eastern Europe | Hungary | IPK | Landrace | GP1 | 1 | 687 | 1707 |
| EUC_GM_078 | Western Europe | Germany | IPK | Variety | GP1 | 1 | 743 | 1770 |
| EUC_GM_079 | Canada | Canada | SG Ceresco | Variety | GP1 | 1 | 892 | 1812 |
| EUC_GM_080 | Canada | Canada | SG Ceresco | Variety | GP1 | 1 | 761 | 1869 |
| EUC_GM_081 | Eastern Europe | Ukraine | IFRAP NAAS | Variety | GP1 | 1 | 697 | 1692 |
| EUC_GM_082 | Western Europe | Germany | USDA-ARS | Breeding line | GP1 | 1 | 859 | 1758 |
| EUC_GM_083 | Western Europe | Germany | USDA-ARS | Breeding line | GP1 | 1 | 779 | 1777 |
| EUC_GM_084 | Western Europe | Germany | USDA-ARS | Landrace | GP1 | 1 | 720 | 1684 |
| EUC_GM_085 | USA | USA | USDA-ARS | Variety | GP1 | 1 | 865 | 1857 |
| EUC_GM_086 | Western Europe | Germany | USDA-ARS | Landrace | GP1 | 1 | 732 | 1651 |
| EUC_GM_087 | Southern Europe | Serbia | IFVCNS | Variety | GP1 | 1 | 914 | 1904 |
| EUC_GM_088 | Eastern Europe | Russia | USDA-ARS | Landrace | GP1 | 1 | 792 | 1782 |
| EUC_GM_089 | Eastern Europe | Bulgaria | USDA-ARS | Landrace | GP1 | 1 | 955 | 1734 |
| EUC_GM_090 | Eastern Europe | CzechRepublic | ART | Variety | GP1 | 1 | 670 | 1709 |
| EUC_GM_091 | Southern Europe | Serbia | IFVCNS | Variety | GP2 | 6 | 889 | 1777 |
| EUC_GM_092 | Canada | Canada | Prograin | Variety | GP2 | 9 | 491 | 1708 |
| EUC_GM_093 | Canada | Canada | Prograin | Variety | GP2 | 6 | 477 | 1654 |
| EUC_GM_094 | Canada | Canada | University of Guelph | Variety | GP2 | 3 | 531 | 1692 |
| EUC_GM_095 | Eastern Europe | Ukraine | IFRAP NAAS | Variety | GP2 | 3 | 605 | 1602 |
| EUC_GM_096 | Southern Europe | Italy | ERSA | Variety | GP2 | 3 | 799 | 1799 |
| EUC_GM_097 | Western Europe | Belgium | Storm Seeds | Breeding line | GP2 | 1 | 533 | 1647 |
| EUC_GM_098 | USA | USA | IPK | Variety | GP2 | 1 | 713 | 1634 |
| EUC_GM_099 | Unknown | Unknown | IFVCNS | Variety | GP2 | 1 | 933 | 1755 |
| EUC_GM_100 | Unknown | Unknown | IFVCNS | Variety | GP2 | 1 | 623 | 1675 |
| EUC_GM_101 | Eastern Europe | CzechRepublic | ART | Variety | GP2 | 1 | 759 | 1743 |
| EUC_GM_102 | Western Europe | Germany | IPK | Variety | GP2 | 1 | 660 | 1572 |
| EUC_GM_103 | Southern Europe | Serbia | IFVCNS | Variety | GP2 | 1 | 810 | 1798 |
| EUC_GM_104 | USA | USA | IFVCNS | Variety | GP2 | 1 | 566 | 1645 |
| EUC_GM_105 | Southern Europe | Serbia | IFVCNS | Variety | GP2 | 1 | 825 | 1754 |
| EUC_GM_106 | Southern Europe | Serbia | IFVCNS | Breeding line | GP2 | 1 | 839 | 1765 |
| EUC_GM_107 | Southern Europe | Serbia | IFVCNS | Breeding line | GP2 | 1 | 876 | 1832 |
| EUC_GM_108 | Southern Europe | Serbia | IFVCNS | Breeding line | GP2 | 1 | 905 | 1837 |
| EUC_GM_109 | Southern Europe | Serbia | IFVCNS | Breeding line | GP2 | 1 | 833 | 1850 |
| EUC_GM_110 | Southern Europe | Serbia | IFVCNS | Breeding line | GP2 | 1 | 904 | 1876 |
| EUC_GM_111 | Unknown | Unknown | IFVCNS | Variety | GP2 | 1 | 904 | 1794 |
| EUC_GM_112 | Canada | Canada | University of Guelph | Variety | GP2 | 1 | 735 | 1768 |
| EUC_GM_113 | Eastern Europe | Ukraine | IFRAP NAAS | Variety | GP2 | 1 | 591 | 1585 |
| EUC_GM_114 | Eastern Europe | Ukraine | IFRAP NAAS | Variety | GP2 | 1 | 641 | 1570 |
| EUC_GM_115 | Eastern Europe | CzechRepublic | ART | Breeding line | GP2 | 1 | 526 | 1620 |
| EUC_GM_116 | Western Europe | Belgium | ILVO | Breeding line | GP2 | 1 | 507 | 1605 |
| EUC_GM_117 | Western Europe | Switzerland | IPK | Variety | GP2 | 1 | 584 | 1562 |
| EUC_GM_118 | Southern Europe | Serbia | MRIZP | Variety | GP2 | 1 | 910 | 1870 |
| EUC_GM_119 | Southern Europe | Serbia | MRIZP | Variety | GP2 | 1 | 914 | 1824 |
| EUC_GM_120 | Southern Europe | Serbia | MRIZP | Breeding line | GP2 | 1 | 873 | 1828 |
| EUC_GM_121 | Western Europe | Belgium | Storm Seeds | Breeding line | GP2 | 1 | 505 | 1630 |
| EUC_GM_122 | Western Europe | Belgium | Storm Seeds | Breeding line | GP2 | 1 | 721 | 1577 |
| EUC_GM_123 | Western Europe | Belgium | Storm Seeds | Breeding line | GP2 | 1 | 505 | 1741 |
| EUC_GM_124 | Western Europe | Belgium | Storm Seeds | Breeding line | GP2 | 1 | 523 | 1639 |
| EUC_GM_125 | Western Europe | Belgium | Storm Seeds | Breeding line | GP2 | 1 | 625 | 1609 |
| EUC_GM_126 | Western Europe | Belgium | Storm Seeds | Breeding line | GP2 | 1 | 767 | 1685 |
| EUC_GM_127 | Unknown | Unknown | IFVCNS | Variety | GP2 | 1 | 838 | 1735 |
| EUC_GM_128 | Unknown | Unknown | IFVCNS | Variety | GP2 | 1 | 869 | 1798 |
| EUC_GM_129 | Canada | Canada | IPK | Variety | GP2 | 1 | 769 | 1686 |
| EUC_GM_130 | Unknown | Unknown | IFVCNS | Variety | GP2 | 1 | 920 | 1787 |
| EUC_GM_131 | Southern Europe | Serbia | IFVCNS | Variety | GP2 | 1 | 787 | 1687 |
| EUC_GM_132 | Southern Europe | Serbia | IFVCNS | Variety | GP2 | 1 | 917 | 1798 |
| EUC_GM_133 | China | China | USDA-ARS | Variety | GP2 | 1 | 581 | 1582 |
| EUC_GM_134 | Southern Europe | Serbia | IFVCNS | Variety | GP2 | 1 | 711 | 1758 |
| EUC_GM_135 | Eastern Europe | Poland | USDA-ARS | Landrace | GP2 | 1 | 585 | 1600 |
| EUC_GM_136 | Western Europe | Switzerland | IPK | Variety | GP2 | 1 | 529 | 1711 |
| EUC_GM_137 | Eastern Europe | Ukraine | IPK | Landrace | GP2 | 1 | 697 | 1607 |
| EUC_GM_138 | USA | USA | IFVCNS | Variety | GP2 | 1 | 559 | 1596 |
| EUC_GM_139 | Canada | Canada | IFVCNS | Variety | GP2 | 1 | 679 | 1608 |
| EUC_GM_140 | Western Europe | France | IPK | Landrace | GP2 | 1 | 565 | 1556 |
| EUC_GM_141 | Unknown | Unknown | IFVCNS | Variety | GP2 | 1 | 610 | 1686 |
| EUC_GM_142 | Western Europe | Germany | Peter Goertz | Breeding line | GP2 | 1 | 574 | 1603 |
| EUC_GM_143 | Eastern Europe | Poland | IPK | Landrace | GP2 | 1 | 577 | 1570 |
| EUC_GM_144 | Southern Europe | Serbia | IFVCNS | Variety | GP2 | 1 | 860 | 1791 |
| EUC_GM_145 | Western Europe | Germany | IPK | Variety | GP2 | 1 | 562 | 1591 |
| EUC_GM_146 | Western Europe | France | IPK | Landrace | GP2 | 1 | 527 | 1665 |
| EUC_GM_147 | Unknown | Unknown | IFVCNS | Breeding line | GP2 | 1 | 812 | 1720 |
| EUC_GM_148 | Southern Europe | Serbia | IFVCNS | Variety | GP2 | 1 | 910 | 1801 |
| EUC_GM_149 | Southern Europe | Serbia | IFVCNS | Variety | GP2 | 1 | 828 | 1803 |
| EUC_GM_150 | Unknown | Unknown | IFVCNS | Breeding line | GP2 | 1 | 850 | 1783 |
| EUC_GM_151 | Eastern Europe | Russia | USDA-ARS | Landrace | GP2 | 1 | 595 | 1592 |
| EUC_GM_152 | Western Europe | France | IPK | Variety | GP2 | 1 | 523 | 1644 |
| EUC_GM_153 | Eastern Europe | Poland | IPK | Variety | GP2 | 1 | 508 | 1694 |
| EUC_GM_154 | Unknown | Unknown | Storm Seeds | Variety | GP2 | 1 | 546 | 1597 |
| EUC_GM_155 | Eastern Europe | Poland | IPK | Landrace | GP2 | 1 | 698 | 1626 |
| EUC_GM_156 | China | China | IPK | Landrace | GP2 | 1 | 663 | 1609 |
| EUC_GM_157 | Unknown | Unknown | IFVCNS | Variety | GP2 | 1 | 682 | 1722 |
| EUC_GM_158 | Southern Europe | Serbia | IPK | Landrace | GP2 | 1 | 488 | 1705 |
| EUC_GM_159 | Southern Europe | Serbia | IFVCNS | Breeding line | GP2 | 1 | 866 | 1802 |
| EUC_GM_160 | Southern Europe | Serbia | IFVCNS | Breeding line | GP2 | 1 | 712 | 1723 |
| EUC_GM_161 | Southern Europe | Serbia | IFVCNS | Breeding line | GP2 | 1 | 904 | 1827 |
| EUC_GM_162 | Unknown | Unknown | IFVCNS | Breeding line | GP2 | 1 | 812 | 1713 |
| EUC_GM_163 | Unknown | Unknown | IFVCNS | Variety | GP2 | 1 | 825 | 1776 |
| EUC_GM_164 | Northern Europe | Belarus | Soya North Co. Ltd. | Variety | GP2 | 1 | 554 | 1578 |
| EUC_GM_165 | Southern Europe | Serbia | IFVCNS | Variety | GP2 | 1 | 884 | 1725 |
| EUC_GM_166 | Canada | Canada | SG Ceresco | Variety | GP2 | 1 | 520 | 1593 |
| EUC_GM_167 | Unknown | Unknown | IFVCNS | Variety | GP2 | 1 | 797 | 1717 |
| EUC_GM_168 | Eastern Europe | Moldova | IPK | Landrace | GP2 | 1 | 646 | 1605 |
| EUC_GM_169 | Western Europe | Germany | USDA-ARS | Breeding line | GP2 | 1 | 684 | 1666 |
| EUC_GM_170 | Southern Europe | Serbia | IFVCNS | Variety | GP2 | 1 | 672 | 1747 |
| EUC_GM_171 | Western Europe | France | IPK | Landrace | GP2 | 1 | 594 | 1572 |
| EUC_GM_172 | Western Europe | Germany | USDA-ARS | Breeding line | GP2 | 1 | 588 | 1640 |
| EUC_GM_173 | Japan | Japan | USDA-ARS | Landrace | GP2 | 1 | 596 | 1681 |
| EUC_GM_174 | Japan | Japan | USDA-ARS | Landrace | GP2 | 1 | 642 | 1698 |
| EUC_GM_175 | Eastern Europe | Romania | IPK | Landrace | GP2 | 1 | 709 | 1807 |
| EUC_GM_176 | Unknown | Unknown | IFVCNS | Variety | GP2 | 1 | 736 | 1683 |
| EUC_GM_177 | Unknown | Unknown | IFVCNS | Variety | GP2 | 1 | 855 | 1762 |
| EUC_GM_178 | Eastern Europe | Hungary | IPK | Landrace | GP2 | 1 | 610 | 1566 |
| EUC_GM_179 | Unknown | Unknown | IFVCNS | Variety | GP2 | 1 | 790 | 1731 |
| EUC_GM_180 | Western Europe | Netherlands | IPK | Breeding line | GP2 | 1 | 589 | 1518 |
| EUC_GM_181 | Canada | Canada | Saatbau Linz | Variety | GP3 | 6 | 631 | 1641 |
| EUC_GM_182 | Southern Europe | Serbia | IFVCNS | Variety | GP3 | 6 | 534 | 1617 |
| EUC_GM_183 | Western Europe | Austria | Saatbau Linz | Variety | GP3 | 6 | 562 | 1614 |
| EUC_GM_184 | Southern Europe | Serbia | IFVCNS | Variety | GP3 | 3 | 625 | 1556 |
| EUC_GM_185 | Unknown | Unknown | ART | Variety | GP3 | 3 | 627 | 1602 |
| EUC_GM_186 | Western Europe | Germany | Saatbau Linz | Variety | GP3 | 3 | 570 | 1628 |
| EUC_GM_187 | Eastern Europe | CzechRepublic | ART | Variety | GP3 | 1 | 568 | 1617 |
| EUC_GM_188 | Western Europe | Austria | ART | Variety | GP3 | 1 | 585 | 1618 |
| EUC_GM_189 | Canada | Canada | Prograin | Variety | GP3 | 1 | 560 | 1700 |
| EUC_GM_190 | Western Europe | Germany | IPK | Variety | GP3 | 1 | 525 | 1512 |
| EUC_GM_191 | Canada | Canada | ART | Variety | GP3 | 1 | 589 | 1585 |
| EUC_GM_192 | Western Europe | Germany | IPK | Variety | GP3 | 1 | 647 | 1619 |
| EUC_GM_193 | Canada | Canada | IFVCNS | Variety | GP3 | 1 | 558 | 1624 |
| EUC_GM_194 | Southern Europe | Serbia | IFVCNS | Variety | GP3 | 1 | 815 | 1650 |
| EUC_GM_195 | Western Europe | Germany | IPK | Variety | GP3 | 1 | 571 | 1553 |
| EUC_GM_196 | Southern Europe | Serbia | IFVCNS | Variety | GP3 | 1 | 595 | 1686 |
| EUC_GM_197 | Unknown | Unknown | IFVCNS | Breeding line | GP3 | 1 | 610 | 1632 |
| EUC_GM_198 | Unknown | Unknown | IFVCNS | Breeding line | GP3 | 1 | 828 | 1719 |
| EUC_GM_199 | Eastern Europe | CzechRepublic | ART | Breeding line | GP3 | 1 | 590 | 1617 |
| EUC_GM_200 | Eastern Europe | CzechRepublic | ART | Variety | GP3 | 1 | 739 | 1605 |
| EUC_GM_201 | Eastern Europe | CzechRepublic | ART | Variety | GP3 | 1 | 720 | 1708 |
| EUC_GM_202 | Canada | Canada | ART | Variety | GP3 | 1 | 507 | 1575 |
| EUC_GM_203 | Eastern Europe | CzechRepublic | ART | Variety | GP3 | 1 | 804 | 1677 |
| EUC_GM_204 | Canada | Canada | IPK | Variety | GP3 | 1 | 583 | 1593 |
| EUC_GM_205 | Eastern Europe | CzechRepublic | ART | Variety | GP3 | 1 | 765 | 1636 |
| EUC_GM_206 | Southern Europe | Serbia | IFVCNS | Breeding line | GP3 | 1 | 787 | 1724 |
| EUC_GM_207 | Eastern Europe | CzechRepublic | ART | Variety | GP3 | 1 | 529 | 1634 |
| EUC_GM_208 | Eastern Europe | CzechRepublic | ART | Variety | GP3 | 1 | 632 | 1635 |
| EUC_GM_209 | Western Europe | Belgium | ILVO | Breeding line | GP3 | 1 | 514 | 1559 |
| EUC_GM_210 | Western Europe | Belgium | ILVO | Breeding line | GP3 | 1 | 503 | 1574 |
| EUC_GM_211 | Canada | Canada | ART | Variety | GP3 | 1 | 516 | 1605 |
| EUC_GM_212 | Eastern Europe | CzechRepublic | ART | Variety | GP3 | 1 | 774 | 1677 |
| EUC_GM_213 | Eastern Europe | CzechRepublic | ART | Breeding line | GP3 | 1 | 748 | 1601 |
| EUC_GM_214 | Canada | Canada | ART | Variety | GP3 | 1 | 556 | 1589 |
| EUC_GM_215 | Canada | Canada | IPK | Variety | GP3 | 1 | 591 | 1537 |
| EUC_GM_216 | Japan | Japan | IPK | Variety | GP3 | 1 | 679 | 1577 |
| EUC_GM_217 | Unknown | Unknown | IFVCNS | Variety | GP3 | 1 | 809 | 1710 |
| EUC_GM_218 | Unknown | Unknown | IFVCNS | Variety | GP3 | 1 | 843 | 1727 |
| EUC_GM_219 | Eastern Europe | CzechRepublic | ART | Landrace | GP3 | 1 | 700 | 1503 |
| EUC_GM_220 | Unknown | Unknown | IFVCNS | Variety | GP3 | 1 | 834 | 1746 |
| EUC_GM_221 | Southern Europe | Serbia | IFVCNS | Variety | GP3 | 1 | 514 | 1639 |
| EUC_GM_222 | Unknown | Unknown | IFVCNS | Variety | GP3 | 1 | 753 | 1639 |
| EUC_GM_223 | Western Europe | Belgium | Storm Seeds | Breeding line | GP3 | 1 | 557 | 1593 |
| EUC_GM_224 | Western Europe | Belgium | Storm Seeds | Breeding line | GP3 | 1 | 834 | 1636 |
| EUC_GM_225 | Western Europe | Belgium | Storm Seeds | Breeding line | GP3 | 1 | 868 | 1642 |
| EUC_GM_226 | Western Europe | Belgium | Storm Seeds | Breeding line | GP3 | 1 | 639 | 1613 |
| EUC_GM_227 | Western Europe | Belgium | Storm Seeds | Breeding line | GP3 | 1 | 670 | 1605 |
| EUC_GM_228 | Western Europe | Belgium | Storm Seeds | Breeding line | GP3 | 1 | 793 | 1607 |
| EUC_GM_229 | Western Europe | Belgium | Storm Seeds | Breeding line | GP3 | 1 | 795 | 1650 |
| EUC_GM_230 | Western Europe | Belgium | Storm Seeds | Breeding line | GP3 | 1 | 609 | 1639 |
| EUC_GM_231 | Western Europe | Belgium | Storm Seeds | Breeding line | GP3 | 1 | 612 | 1611 |
| EUC_GM_232 | Western Europe | Belgium | Storm Seeds | Breeding line | GP3 | 1 | 589 | 1595 |
| EUC_GM_233 | Western Europe | Belgium | Storm Seeds | Breeding line | GP3 | 1 | 738 | 1674 |
| EUC_GM_234 | Western Europe | Belgium | Storm Seeds | Breeding line | GP3 | 1 | NA | NA |
| EUC_GM_235 | Western Europe | Belgium | Storm Seeds | Breeding line | GP3 | 1 | 564 | 1597 |
| EUC_GM_236 | Western Europe | Belgium | Storm Seeds | Breeding line | GP3 | 1 | 529 | 1639 |
| EUC_GM_237 | Western Europe | Belgium | Storm Seeds | Breeding line | GP3 | 1 | 559 | 1548 |
| EUC_GM_238 | Canada | Canada | SG Ceresco | Variety | GP3 | 1 | 505 | 1485 |
| EUC_GM_239 | Canada | Canada | IFVCNS | Variety | GP3 | 1 | 791 | 1640 |
| EUC_GM_240 | Eastern Europe | Poland | IPK | Landrace | GP3 | 1 | 688 | 1616 |
| EUC_GM_241 | Western Europe | Germany | IFVCNS | Variety | GP3 | 1 | 718 | 1575 |
| EUC_GM_242 | Unknown | Unknown | IFVCNS | Breeding line | GP3 | 1 | 480 | 1535 |
| EUC_GM_243 | Unknown | Unknown | IFVCNS | Breeding line | GP3 | 1 | 727 | 1669 |
| EUC_GM_244 | Western Europe | France | USDA-ARS | Landrace | GP3 | 1 | 656 | 1536 |
| EUC_GM_245 | Western Europe | France | USDA-ARS | Landrace | GP3 | 1 | 609 | 1578 |
| EUC_GM_246 | Western Europe | Germany | Peter Goertz | Breeding line | GP3 | 1 | 592 | 1707 |
| EUC_GM_247 | Southern Europe | Serbia | IFVCNS | Variety | GP3 | 1 | 849 | 1667 |
| EUC_GM_248 | Western Europe | Switzerland | IPK | Variety | GP3 | 1 | 566 | 1541 |
| EUC_GM_249 | Canada | Canada | IPK | Variety | GP3 | 1 | 657 | 1547 |
| EUC_GM_250 | Eastern Europe | CzechRepublic | ART | Landrace | GP3 | 1 | 859 | 1757 |
| EUC_GM_251 | Western Europe | Germany | IPK | Variety | GP3 | 1 | 599 | 1556 |
| EUC_GM_252 | Eastern Europe | Moldova | IPK | Landrace | GP3 | 1 | 618 | 1540 |
| EUC_GM_253 | Unknown | Unknown | IFVCNS | Variety | GP3 | 1 | 691 | 1599 |
| EUC_GM_254 | Canada | Canada | IFVCNS | Variety | GP3 | 1 | 470 | 1567 |
| EUC_GM_255 | Canada | Canada | USDA-ARS | Variety | GP3 | 1 | 483 | 1502 |
| EUC_GM_256 | Unknown | Unknown | IFVCNS | Breeding line | GP3 | 1 | 597 | 1621 |
| EUC_GM_257 | Southern Europe | Serbia | IFVCNS | Variety | GP3 | 1 | 709 | 1613 |
| EUC_GM_258 | Unknown | Unknown | IFVCNS | Breeding line | GP3 | 1 | 686 | 1533 |
| EUC_GM_259 | Southern Europe | Serbia | IFVCNS | Variety | GP3 | 1 | 779 | 1704 |
| EUC_GM_261 | Unknown | Unknown | IFVCNS | Variety | GP3 | 1 | 817 | 1636 |
| EUC_GM_262 | Western Europe | Belgium | ILVO | Breeding line | GP3 | 1 | 552 | 1552 |
| EUC_GM_263 | Western Europe | Belgium | ILVO | Breeding line | GP3 | 1 | 616 | 1591 |
| EUC_GM_264 | Western Europe | Belgium | ILVO | Breeding line | GP3 | 1 | 502 | 1686 |
| EUC_GM_266 | Eastern Europe | CzechRepublic | ART | Breeding line | GP3 | 1 | 529 | 1576 |
| EUC_GM_267 | Eastern Europe | Russia | IPK | Landrace | GP3 | 1 | 709 | 1485 |
| EUC_GM_268 | Eastern Europe | CzechRepublic | ART | Breeding line | GP3 | 1 | 547 | 1612 |
| EUC_GM_269 | Unknown | Unknown | IFVCNS | Variety | GP3 | 1 | 914 | 1707 |
| EUC_GM_270 | Eastern Europe | Bulgaria | IPK | Landrace | GP3 | 1 | 722 | 1539 |
| EUC_GM_271 | Northern Europe | Sweden | NordGen | Variety | GP4 | 6 | 636 | 1476 |
| EUC_GM_272 | Western Europe | Austria | Saatbau Linz | Variety | GP4 | 9 | 600 | 1592 |
| EUC_GM_273 | Eastern Europe | Russia | USDA-ARS | Variety | GP4 | 6 | 566 | 1421 |
| EUC_GM_274 | Western Europe | Austria | ART | Variety | GP4 | 3 | 635 | 1592 |
| EUC_GM_275 | Canada | Canada | Saatbau Linz | Variety | GP4 | 3 | 660 | 1632 |
| EUC_GM_276 | Northern Europe | Belarus | Soya North Co. Ltd. | Variety | GP4 | 3 | 596 | 1548 |
| EUC_GM_277 | Western Europe | Germany | USDA-ARS | Breeding line | GP4 | 1 | 785 | 1523 |
| EUC_GM_278 | Western Europe | Belgium | Storm Seeds | Breeding line | GP4 | 1 | 721 | 1537 |
| EUC_GM_279 | Western Europe | Belgium | Storm Seeds | Breeding line | GP4 | 1 | 774 | 1556 |
| EUC_GM_280 | Western Europe | Belgium | Storm Seeds | Breeding line | GP4 | 1 | 723 | 1558 |
| EUC_GM_281 | Western Europe | Belgium | Storm Seeds | Breeding line | GP4 | 1 | 781 | 1597 |
| EUC_GM_282 | Eastern Europe | Poland | IPK | Variety | GP4 | 1 | 730 | 1536 |
| EUC_GM_283 | Canada | Canada | IPK | Variety | GP4 | 1 | 643 | 1505 |
| EUC_GM_284 | Canada | Canada | ART | Variety | GP4 | 1 | 584 | 1565 |
| EUC_GM_285 | Northern Europe | Sweden | NordGen | Variety | GP4 | 1 | 557 | 1488 |
| EUC_GM_286 | Unknown | Unknown | IFVCNS | Breeding line | GP4 | 1 | 685 | 1573 |
| EUC_GM_287 | Unknown | Unknown | IFVCNS | Variety | GP4 | 1 | 983 | 1741 |
| EUC_GM_288 | Western Europe | Germany | USDA-ARS | Breeding line | GP4 | 1 | 812 | 1622 |
| EUC_GM_289 | Eastern Europe | Ukraine | IPK | Landrace | GP4 | 1 | 735 | 1539 |
| EUC_GM_290 | Western Europe | Germany | IPK | Variety | GP4 | 1 | 766 | 1482 |
| EUC_GM_291 | Western Europe | Belgium | Storm Seeds | Breeding line | GP4 | 1 | 701 | 1517 |
| EUC_GM_292 | Western Europe | Belgium | Storm Seeds | Breeding line | GP4 | 1 | 708 | 1639 |
| EUC_GM_293 | Western Europe | Belgium | Storm Seeds | Breeding line | GP4 | 1 | 718 | 1657 |
| EUC_GM_294 | Western Europe | Belgium | Storm Seeds | Breeding line | GP4 | 1 | 650 | 1537 |
| EUC_GM_295 | Western Europe | Belgium | Storm Seeds | Breeding line | GP4 | 1 | 592 | 1592 |
| EUC_GM_296 | Western Europe | Belgium | Storm Seeds | Breeding line | GP4 | 1 | 679 | 1655 |
| EUC_GM_297 | Western Europe | Belgium | Storm Seeds | Breeding line | GP4 | 1 | 707 | 1616 |
| EUC_GM_298 | Western Europe | Belgium | Storm Seeds | Breeding line | GP4 | 1 | 752 | 1578 |
| EUC_GM_299 | Western Europe | Belgium | Storm Seeds | Breeding line | GP4 | 1 | 824 | 1617 |
| EUC_GM_300 | Western Europe | Belgium | Storm Seeds | Breeding line | GP4 | 1 | 727 | 1619 |
| EUC_GM_301 | Western Europe | Belgium | Storm Seeds | Breeding line | GP4 | 1 | 566 | 1644 |
| EUC_GM_302 | Western Europe | Belgium | Storm Seeds | Breeding line | GP4 | 1 | 679 | 1616 |
| EUC_GM_303 | Western Europe | Belgium | Storm Seeds | Breeding line | GP4 | 1 | 655 | 1627 |
| EUC_GM_304 | Western Europe | Belgium | Storm Seeds | Breeding line | GP4 | 1 | 656 | 1594 |
| EUC_GM_305 | Western Europe | Belgium | Storm Seeds | Breeding line | GP4 | 1 | 604 | 1576 |
| EUC_GM_306 | Eastern Europe | Romania | USDA-ARS | Breeding line | GP4 | 1 | 713 | 1547 |
| EUC_GM_307 | Northern Europe | Sweden | USDA-ARS | Breeding line | GP4 | 1 | 628 | 1486 |
| EUC_GM_308 | Northern Europe | Sweden | USDA-ARS | Variety | GP4 | 1 | 618 | 1522 |
| EUC_GM_309 | Western Europe | Germany | USDA-ARS | Breeding line | GP4 | 1 | 891 | 1631 |
| EUC_GM_310 | Western Europe | Austria | Saatzucht Gleisdorf | Variety | GP4 | 1 | 538 | 1620 |
| EUC_GM_311 | Japan | Japan | IPK | Landrace | GP4 | 1 | 709 | 1447 |
| EUC_GM_312 | Eastern Europe | Russia | IPK | Landrace | GP4 | 1 | 600 | 1534 |
| EUC_GM_313 | Western Europe | Germany | Peter Goertz | Breeding line | GP4 | 1 | 746 | 1601 |
| EUC_GM_314 | Western Europe | Germany | Peter Goertz | Breeding line | GP4 | 1 | 742 | 1586 |
| EUC_GM_315 | Unknown | Unknown | IFVCNS | Variety | GP4 | 1 | 808 | 1576 |
| EUC_GM_316 | Western Europe | France | IPK | Landrace | GP4 | 1 | 889 | 1547 |
| EUC_GM_317 | Eastern Europe | Russia | USDA-ARS | Landrace | GP4 | 1 | 709 | 1573 |
| EUC_GM_318 | Japan | Japan | IPK | Landrace | GP4 | 1 | 635 | 1472 |
| EUC_GM_319 | Eastern Europe | Russia | NordGen | Variety | GP4 | 1 | 569 | 1426 |
| EUC_GM_320 | Eastern Europe | Russia | IPK | Variety | GP4 | 1 | 631 | 1536 |
| EUC_GM_321 | Eastern Europe | CzechRepublic | ART | Variety | GP4 | 1 | 845 | 1655 |
| EUC_GM_322 | Northern Europe | Estonia | ECRI | Variety | GP4 | 1 | 683 | 1468 |
| EUC_GM_323 | Unknown | Unknown | IFVCNS | Variety | GP4 | 1 | 775 | 1597 |
| EUC_GM_324 | Northern Europe | Lithuania | IPK | Landrace | GP4 | 1 | 796 | 1519 |
| EUC_GM_325 | Eastern Europe | Russia | USDA-ARS | Variety | GP4 | 1 | 618 | 1448 |
| EUC_GM_326 | Canada | Canada | IPK | Variety | GP4 | 1 | 749 | 1587 |
| EUC_GM_327 | Canada | Canada | IPK | Variety | GP4 | 1 | 625 | 1533 |
| EUC_GM_328 | Canada | Canada | IPK | Variety | GP4 | 1 | 733 | 1582 |
| EUC_GM_329 | Unknown | Unknown | IFVCNS | Variety | GP4 | 1 | 714 | 1600 |
| EUC_GM_330 | Canada | Canada | ART | Variety | GP4 | 1 | 591 | 1557 |
| EUC_GM_331 | Unknown | Unknown | IFVCNS | Variety | GP4 | 1 | 605 | 1519 |
| EUC_GM_332 | Eastern Europe | Poland | IPK | Variety | GP4 | 1 | 639 | 1499 |
| EUC_GM_333 | Eastern Europe | Poland | IPK | Variety | GP4 | 1 | 621 | 1545 |
| EUC_GM_334 | Eastern Europe | Poland | IPK | Variety | GP4 | 1 | 636 | 1547 |
| EUC_GM_335 | Northern Europe | Belarus | Soya North Co. Ltd. | Variety | GP4 | 1 | 592 | 1540 |
| EUC_GM_336 | Eastern Europe | Poland | IPK | Variety | GP4 | 1 | 671 | 1528 |
| EUC_GM_337 | Unknown | Unknown | ART | Variety | GP4 | 1 | 623 | 1526 |
| EUC_GM_338 | Western Europe | Belgium | ILVO | Breeding line | GP4 | 1 | 609 | 1580 |
| EUC_GM_339 | Western Europe | Belgium | ILVO | Breeding line | GP4 | 1 | 653 | 1549 |
| EUC_GM_340 | Western Europe | Belgium | ILVO | Breeding line | GP4 | 1 | 642 | 1539 |
| EUC_GM_341 | Western Europe | Belgium | ILVO | Breeding line | GP4 | 1 | 625 | 1534 |
| EUC_GM_342 | Japan | Japan | IPK | Variety | GP4 | 1 | 628 | 1488 |
| EUC_GM_343 | Japan | Japan | IPK | Variety | GP4 | 1 | 725 | 1579 |
| EUC_GM_344 | Western Europe | Germany | IPK | Variety | GP4 | 1 | 574 | 1435 |
| EUC_GM_345 | Western Europe | Germany | IPK | Landrace | GP4 | 1 | 763 | 1594 |
| EUC_GM_346 | Western Europe | Germany | USDA-ARS | Breeding line | GP4 | 1 | 741 | 1576 |
| EUC_GM_347 | Northern Europe | Sweden | NordGen | Variety | GP4 | 1 | 585 | 1431 |
| EUC_GM_348 | Unknown | Unknown | IFVCNS | Variety | GP4 | 1 | 675 | 1681 |
| EUC_GM_349 | Northern Europe | Sweden | IPK | Breeding line | GP4 | 1 | 689 | 1465 |
| EUC_GM_350 | Northern Europe | Lithuania | IPK | Landrace | GP4 | 1 | 760 | 1534 |
| EUC_GM_351 | Unknown | Unknown | IFVCNS | Variety | GP4 | 1 | 891 | 1725 |
| EUC_GM_352 | Northern Europe | Belarus | Soya North Co. Ltd. | Variety | GP4 | 1 | 754 | 1542 |
| EUC_GM_353 | Eastern Europe | Russia | IPK | Landrace | GP4 | 1 | 725 | 1535 |
| EUC_GM_354 | Northern Europe | Sweden | USDA-ARS | Breeding line | GP4 | 1 | 757 | 1532 |
| EUC_GM_355 | Northern Europe | Sweden | USDA-ARS | Breeding line | GP4 | 1 | 558 | 1476 |
| EUC_GM_356 | China | China | IPK | Landrace | GP4 | 1 | 762 | 1542 |
| EUC_GM_357 | Eastern Europe | Russia | IPK | Landrace | GP4 | 1 | 611 | 1490 |
| EUC_GM_358 | China | China | IPK | Landrace | GP4 | 1 | 801 | 1567 |
| EUC_GM_359 | Western Europe | Germany | USDA-ARS | Breeding line | GP4 | 1 | 784 | 1530 |
| EUC_GM_360 | Eastern Europe | Russia | USDA-ARS | Variety | GP4 | 1 | 761 | 1532 |
| EUC_GM_441 | Eastern Europe | Russia | USDA-ARS | Landrace | GP2 | 3 | 770 | 1735 |

*Source of Accessions:

IFVCNS: Institute of Field and Vegetable Crops, Novisad Serbia

USDA-ARS: U.S. Department of Agriculture ARS-GRIN database of germplasm resources

ERSA: Agenzia regionale per lo Sviluppo Rurale del Friuli Venezia Giulia, Italy

NARDI Fundulea: National Agricultural Research and Development Institue, Romania

SCDA Turda: Statiunea de Cercetare Dezvoltare Agricola Turda, Romania

IFRAP NAAS: Institute of Feed research and Agriculture of Podillya of NAAS, Ukraine

ART: Agricultural Research, LTd. (ART), SME Czech Republic

IFC-Pleven : Institute of Forage Crops, Pleven Bulgaria

MRIZP: Maize Research Institute Zemun Polje, Belgrade Serbia

IPK: Leibniz Institute of Plant Genetics and Crop Plant Research, Germaany

Storm Seeds: Agro Seeds Service bvba, Belgium

SG Ceresco: SG Ceresco, Canada

Prograin: Prograin, Canada

ILVO: Instituut voor Landbouw, Vissserij en Voedingsonderzoek, Melle Belgium

Saatbau Linz: Saatbau Linz, Germany

NordGen: The Nordic Genetic Resource Center, Sweden

Saatzucht Gleisdorf: Saatzucht Gleisdorf, Austria

ECRI: Estonian Crop Research Institute, Estonia

Soya North Co. Ltd.: Soya North Co. Ltd., Belarus

**Table S2.** Description of the field management activities in 2018 and 2019.

| Activity | Description | Year | |
| --- | --- | --- | --- |
|  |  | 2018 | 2019 |
| Inoculation of seeds | Commercial strain of Bradyrhizobium japonicum BIODOZ® (De Sangosse, France) in 2018 and HiStick® (BASF, USA) in 2019 at a rate of 400 g /100 g of seed. Inoculum was prepared in ¾ liter water and mixed with the seeds. | Pre-sowing | Pre-sowing |
| Fertilization | Single application of 36-28-140 kg ha-1 N–P–K. | Pre-sowing | Pre-sowing |
| Sowing | Manually at four different moments. | 20 April (GP1), 3 May (GP2), 8 May (GP3), 11 May (GP4) | 19 April (GP1), 30 April (GP2), 10 May (GP3), 15 May (GP4) |
| Herbicide treatment | Single application of STOMP ACQUA (BASF, Germany) at a rate of 0.91 kg ha-1 and Centium 360 CS (FMC, The Netherlands) at a rate of 0.72 kg ha-1. | Pre-emergence | Pre-emergence |
| Weeding | Manually with the help of garden hoe. | After emergence | After emergence |
| Thinning | Extra seedlings removed after emergence to standardize density in GP1 and GP2 plots. | 24-May | 7-Jun |
| Irrigation | Manual irrigation through hose at seedling stage and sprinkler irrigation at later stages. | See materials and methods | See materials and methods |
| Drought treatment | Complete restriction of water through rainout shelters in drought treatment field. | 22 June to 17 July | 3 July to 21 August |
| Insecticide treatment | Floramite 240 SC (UPL Holdings Coöperatief U.A., The Netherlands) at a rate of 0.4 L ha-1 to control spider mites. | 1 time in July | 1 time in July |
| Harvesting | Five plants in the middle row per plot. | 28 August to 29 October | 3 September to 5 November |

**Table S3.** Summary of model fit results. ‘nObs’ is number of observations after removal of plots with E<30% and outliers; ‘Treat’ is the treatment; ‘Trans’ indicates whether a transformation was applied; ‘Best fit’ is the best fitted model from the six derived versions of the base model; and ‘R^2^’ is the coefficient of determination. CW and LSEN were not determined in the control fields.

| Group | Variable^a^ | Treat^b^ | 2018 | | | | 2019 | | | |
| --- | --- | --- | --- | --- | --- | --- | --- | --- | --- | --- |
|  |  |  | nObs | Trans | Best fit | R^2^ | nObs | Trans | Best fit | R^2^ |
| Derived from manual measurements/ visual scores | PLV | C | 418 |  | Y ~ Genotype + Column | 0.76 | 417 |  | Y ~ Genotype + Column + Row | 0.66 |
|  |  | D | 417 |  | Y ~ Genotype | 0.81 | 381 |  | Y ~ Genotype + Row | 0.66 |
|  | R2 | C | 424 |  | Y ~ Genotype + Block | 0.82 | 425 |  | Y ~ Genotype + Column + Row | 0.97 |
|  |  | D | 423 |  | Y ~ Genotype + Row | 0.92 | 388 |  | Y ~ Genotype | 0.95 |
|  | R5 | C | 408 |  | Y ~ Genotype + Column + Row | 0.76 | 399 |  | Y ~ Genotype + Block + Row | 0.92 |
|  |  | D | 410 |  | Y ~ Genotype + Row | 0.77 | 387 |  | Y ~ Genotype + Column | 0.91 |
|  | R8 | C | 424 |  | Y ~ Genotype + Column + Row | 0.87 | 425 |  | Y ~ Genotype + Column + Row | 0.94 |
|  |  | D | 423 |  | Y ~ Genotype + Row | 0.87 | 378 |  | Y ~ Genotype + Block + Row | 0.79 |
|  | CW | D | 420 |  | Y ~ Genotype + Column + Row | 0.57 | 388 |  | Y ~ Genotype + Column + Row | 0.61 |
|  | LSEN | D | 423 |  | Y ~ Genotype + Row | 0.72 | 378 |  | Y ~ Genotype + Column + Row | 0.61 |
|  | PPS | C | 419 |  | Y ~ Genotype + Column | 0.51 | 416 |  | Y ~ Genotype + Column + Row | 0.62 |
|  |  | D | 418 |  | Y ~ Genotype + Block | 0.73 | 375 |  | Y ~ Genotype + Row | 0.48 |
|  | SN | C | 420 | sqrt | Y ~ Genotype + Column + Row | 0.71 | 406 | sqrt | Y ~ Genotype + Column + Row | 0.52 |
|  |  | D | 417 | sqrt | Y ~ Genotype + Row | 0.87 | 377 | sqrt | Y ~ Genotype + Block + Row | 0.28 |
|  | SW | C | 420 | log | Y ~ Genotype + Column + Row | 0.71 | 409 | log | Y ~ Genotype + Column + Row | 0.52 |
|  |  | D | 419 | log | Y ~ Genotype + Row | 0.88 | 375 | log | Y ~ Genotype | 0.16 |
| UAV-RGB | CC75 | C | 374 |  | Y ~ Genotype + Column + Row | 0.61 | 386 |  | Y ~ Genotype + Block + Row | 0.68 |
|  |  | D | 299 |  | Y ~ Genotype + Row | 0.78 | 349 |  | Y ~ Genotype + Block + Row | 0.58 |
|  | AGRmax | C | 420 |  | Y ~ Genotype + Column + Row | 0.69 | 410 |  | Y ~ Genotype + Column + Row | 0.55 |
|  |  | D | 401 |  | Y ~ Genotype + Column + Row | 0.31 | 377 | log | Y ~ Genotype + Column + Row | 0.54 |
|  | CH | C | 406 |  | Y ~ Genotype + Column + Row | 0.65 | 395 |  | Y ~ Genotype + Column + Row | 0.74 |
|  |  | D | 372 |  | Y ~ Genotype + Column + Row | 0.85 | 355 |  | Y ~ Genotype + Block + Row | 0.61 |
|  | DET | C | 405 |  | Y ~ Genotype + Block | 0.68 | 396 |  | Y ~ Genotype + Block | 0.74 |
|  |  | D | 369 |  | Y ~ Genotype + Row | 0.46 | 365 |  | Y ~ Genotype + Column + Row | 0.45 |
|  | SNC | C | 387 |  | Y ~ Genotype + Column + Row | 0.73 | 389 |  | Y ~ Genotype + Row | 0.91 |
|  |  | D | 299 |  | Y ~ Genotype + Column + Row | 0.71 | 353 |  | Y ~ Genotype + Block + Row | 0.81 |
| UAV-Thermal | CWSI | C | 423 |  | Y ~ Genotype + Column + Row | 0.66 | 420 |  | Y ~ Genotype + Column + Row | 0.52 |
|  |  | D | 421 |  | Y ~ Genotype + Row | 0.2 | 387 |  | Y ~ Genotype + Block | 0.78 |

^a^ For a full description of the variables, see Table 1

^b^ ‘C’ represents the control treatment and ‘D’ represents the drought treatment

**Table S4.** Environmental conditions during different periods in the growing seasons considered in this study. 'T' is the average temperature of the day, 'RH' is the average relative humidity of the day and 'SWR ' is the average of the sum of daily solar short wave radiation. Values between brackets are the minimum and the maximum values for the corresponding period.

| Period* | Year | Period | T (°C) | RH (%) | SWR (MJ m^-2^ day^-1^) |
| --- | --- | --- | --- | --- | --- |
| Sowing | 2018 | 10 Apr to 11 May | 13.8 (7.4, 19.9) | 70.8 (46.5, 94.6) | 21.6 (3.8, 30.2) |
|  | 2019 | 9 Apr to 15 May | 11.8 (6.7, 17.7) | 71.0 (46.0, 91.2) | 18.8 (9.4, 30.4) |
| Vegetative | 2018 | 10 Apr to 17 Jun | 16.1 (10.5, 22.1) | 75.7 (51.1, 95.7) | 21.0 (3.8, 31.7) |
|  | 2019 | 9 Apr to 22 Jun | 13.2( 8.3, 18.6) | 74.8 (52.5, 92.9) | 18.5 (0.8, 31.4) |
| Reproductive | 2018 | 17 Jun to 26 Aug | 20.2 (13.9, 26.6) | 68.3 (42.8, 93.1) | 23.0 (5.5, 33.8) |
|  | 2019 | 22 Jun to 2 Sep | 17.7 (11.9, 24.0) | 73.1 (49.2, 92.8) | 19.9 (1.5, 33.5) |
| Harvesting | 2018 | 26 Aug to 29 Oct | 14.2 (9.1, 20.2) | 80.0 (54.7, 97.0) | 12.6 (2.6, 23.1) |
|  | 2019 | 2 Sep to 5 Nov | 12.4 (8.6, 16.8) | 84.2 (66.9, 94.2) | 8.9 (1.8, 22.4) |

*’Vegetative’ is Period between first sowing and achievement of R1 stage in 50% of the plots, ‘Reproductive’ is Period between R1 stage in 50% of the plots and R8 stage in 50% of the plots and ‘Harvesting’ is Period between R8 stage in 50% of the plots and the last harvest
